# Patient-centered empirical research on ethically relevant psychosocial and cultural aspects of cochlear, glaucoma and cardiovascular implants – a scoping review

**DOI:** 10.1186/s12910-023-00945-6

**Published:** 2023-08-28

**Authors:** Sabine Schulz, Laura Harzheim, Constanze Hübner, Mariya Lorke, Saskia Jünger, Christiane Woopen

**Affiliations:** 1 Cologne Center for Ethics, Rights, Economics, and Social Sciences of Health (CERES), University of Cologne and University Hospital of Cologne, Universitätsstraße 91, 50931 Cologne, Germany; 2 Center for Life Ethics, University of Bonn, 53113 Bonn, Germany; 3 Faculty of Engineering and Mathematics, University of Applied Sciences and Arts (HSBI), 33619 Bielefeld, Germany; 4 Department of Community Health, University of Applied Health Sciences Bochum, Gesundheitscampus 6-8, 44801 Bochum, Germany

**Keywords:** Autonomy, Freedom, Privacy, Safety, Identity, Participation, Justice, Sustainability, Implant ethics, Patient perspective

## Abstract

**Background:**

The significance of medical implants goes beyond technical functioning and reaches into everyday life, with consequences for individuals as well as society. Ethical aspects associated with the everyday use of implants are relevant for individuals’ lifeworlds and need to be considered in implant care and in the course of technical developments.

**Methods:**

This scoping review aimed to provide a synthesis of the existing evidence regarding ethically relevant psychosocial and cultural aspects in cochlear, glaucoma and cardiovascular implants in patient-centered empirical research. Systematic literature searches were conducted in EBSCOhost, Philpapers, PsycNET, Pubmed, Web of Science and BELIT databases. Eligible studies were articles in German or English language published since 2000 dealing with ethically relevant aspects of cochlear, glaucoma and passive cardiovascular implants based on empirical findings from the perspective of (prospective) implant-wearers and their significant others. Following a descriptive-analytical approach, a data extraction form was developed and relevant data were extracted accordingly. We combined a basic numerical analysis of study characteristics with a thematically organized narrative synthesis of the data.

**Results:**

Sixty-nine studies were included in the present analysis. Fifty were in the field of cochlear implants, sixteen in the field of passive cardiovascular implants and three in the field of glaucoma implants. Implant-related aspects were mainly found in connection with *autonomy*, *freedom*, *identity*, *participation* and *justice,* whereas little to no data was found with regards to ethical principles of *privacy, safety* or *sustainability.*

**Conclusions:**

Empirical research on ethical aspects of implant use in everyday life is highly relevant, but marked by ambiguity and unclarity in the operationalization of ethical terms and contextualization. A transparent orientation framework for the exploration and acknowledgment of ethical aspects in “lived experiences” may contribute to the improvement of individual care, healthcare programs and research quality in this area. Ethics-sensitive care requires creating awareness for cultural and identity-related issues, promoting health literacy to strengthen patient autonomy as well as adjusting healthcare programs accordingly. More consideration needs to be given to sustainability issues in implant development and care according to an approach of ethics-by-design.

**Supplementary Information:**

The online version contains supplementary material available at 10.1186/s12910-023-00945-6.

## Introduction

Implant-based interventions for the effective treatment of health conditions like hearing loss, glaucoma or cardiovascular disease have become increasingly advanced. Cochlear implants (CI) enable persons with hearing loss to regain a sense of hearing; through continuous development candidacy criteria have expanded over the years [1]. Children under 6 months can have a device implanted [2]. Regarding the management of glaucoma, minimally invasive glaucoma surgery (MIGS) technology and implants (GI) offer improved safety and effectively reduce intraocular pressure [3]. Passive cardiovascular implants (CVI) like coronary stents or valve implants effectively treat cardiovascular diseases. Innovations like e.g. the Transcatheter Aortic Valve Implantation (TAVI) allow for treatment of individuals who are at risk of complications from open-heart surgery with well-studied short-term results [4]. The current review was part of a collaborative project^1^ conducted in Germany which examined the potentials of innovative medical technology for patients and the healthcare system, emphasizing the long-term perspective of the implant treatment. The focus herein was on the distinctive medical application fields of cardiovascular diseases, glaucoma as well as hearing loss and deafness due to the high epidemiological prevalence on the one hand, and a strong technology-driven need for innovation on the other hand, combined with high market volumes and above-average growth rates on a global perspective [5]. Since in the case of innovative treatments long-term data are hardly available, the use of implants in medicine needs to be assessed both from a medical and technological point of view. Moreover, when developing and implementing innovative technologies, incorporating the analysis of (long-term) ethical issues is an integral part of technology assessment processes [6]. For example, the age range of implantation in conjunction with an increasingly aging population reveals new ethically relevant aspects of implant use related to sustainability (e.g. durability, functionality), care (e.g. easy constant maintenance and adaptation) and technical development (e.g. replacement), all of which should be considered on an individual and societal level. Such aspects are not only relevant in the context of the medical consultation, but also impact individual’s everyday life experiences and identity in the long-term. The ethical debates in the field of CI e.g. goes back to the 1990s and address fundamental conceptions of deafness as a disability or as a culture demonstrating the close intertwining of individual, cultural, medical and technology-related values and understandings [7–10].

Considering the maintenance or restoration of individual health and the sense of hearing or seeing, these implants have a significance beyond altering physical conditions. Implant-based interventions boast (1) cultural/societal and (2) psychosocial aspects, infiltrating individual’s lives and social values:

1. Medical technological developments mirror and shape societal values and convictions on the functionality and social desirability of a human body, health conditions, and treatment decisions [11], potentially leading to the re-negotiation of associated values and norms in a given culture. This interconnectedness between society, culture and (bio-)technologies [12, 13] transpires in the *age of enhancement*, with Western medicine focusing on optimizing human life [14] and in the debates on *cyborgs* or medical consumerism [15].
2. Implant-based interventions modify the body, eliciting changes in functional and social (in)abilities. Thereby, they affect psychosocial aspects of individual’s lifeworlds, e.g. by transforming the way someone reflexively understands themselves [16], or relates to others [17–19].

Some of these aspects are related to fundamental ethical values like autonomy or participation, warranting the examination of ethical relevancies of implant-related aspects in implant-wearer’s lives. For future implant development, a consideration of ethical values informed by empirical research can help to deepen the understanding of living with an implant and to improve the quality of implant care.

### Conceptual frame

In this review, we refer to the ethical approach of *fundamental ethical values* for three reasons: it (1) is closely related to human rights, (2) suits legal perspectives on medical technology innovations (such as implants) and (3) allows for examination and systematization of empirical data.

Human rights declare all human beings to be equal in rights, dignity, and worth [20, 21]. *Health* is defined not merely as freedom from disease and infirmity but encompasses “the state of complete physical, mental, and social well-being “ (p. 1315) [22]. The condition of health reflects the implementation of many other human rights and ethical values [20] like *autonomy*, *self-determination*, *freedom*, *privacy*, *safety, identity*, *participation, justice*, and *sustainability*. In the following, these values will be introduced and their meaning in the context of implants as well as why they determine the conceptual frame of this scoping review will be outlined.

*Autonomy* refers to the basic human ability to “exchange reasons for actions with other individuals [in sensible reasoning] and make responsible decisions on their own initiative. This ability marks humans as moral beings” (p. 38) [23]. *Self-determination* relates to the possibility of realizing one’s own actions and decisions [23], demonstrating an elementary expression of freedom [24]. Understanding the nature of essential aspects that guide a decision presents a prerequisite of self-determination [23]. The patient’s autonomy in DM processes in healthcare is considered an ethical imperative [25]. Autonomy and self-determination play an important role with regards to the decision-making (DM) for or against implant treatment, which is often motivated by the wish for and can contribute to a more autonomous and self-determined way of life. In literature on implant-wearers’ lived experiences the terms *autonomy* and *self-determination* are sometimes used synonymously or with different meanings. Since the majority of studies do not provide exact definitions and *autonomy* is more common in the Anglo-American sphere, it is also used in the current scoping review.

*Freedom* entails the ability to act without internal or external constraints with sufficient resources to effectively implement one’s desires [26]. Freedom and liberty are mainly used interchangeably [27], whereas a distinction is made between negative liberty (absence of obstacles, barriers, constraints or interference from others) and positive liberty ((the possibility of) taking control of one’s life, realizing one’s fundamental purpose, presupposing) [27]. An implant touches on freedom by enabling individuals to pursue their own objectives, influencing outside constraints, or imposing limitations on the implant-wearer itself.

The influence of medical interventions on personal *identity* and moral implications of potential changes are highly contested [28–31], also elaborating on personhood, continuity of psychological identity or the conceptual differentiation from personality [31, 32]. This also applies to medical implants. Changing the implant-wearer’s bodily abilities might affect personality [28] or personal identity (individual goals, values, and beliefs) [29]. The implantation of an implant into the body furthermore touches on the right to physical and mental integrity [21] and relates to aspects of embodiment (cultural, social, physical, psychological, and experiential nature of being a body) [33]. Against this background, the effects of the implant on the implant-wearer’s self-relation and associated aspects (e.g., self-acceptance, self-confidence) are conceivable. Wearing an implant impacts social and cultural identity, meaning the sense of belonging to a group. Here, social identity refers to any group to which a person belongs, whereas cultural identity refers specifically to cultural groups [29].

*Privacy* preserves an individual’s freedom and the integrity of one’s personal identity [24]. Informational privacy refers to a person’s control over their personal information [34]. We focus on device- or implant-related privacy, which is also related to the privacy of one’s health condition, e.g., in the case of (health-)data-processing device components of CIs that can flow into a data system accessible for other people. Moreover, persons with visible implants and therefore ‘externally visible’ diseases or disabilities can be evidently perceptible and tangible for their environment making it difficult for individuals to keep information regarding personal health conditions private.

*Safety* encompasses an individual’s right to a set of certain safety standards (e.g. safe living environment) [35]. Implants can help implant-wearers to live more safely on a very practical level, e.g. by enabling them to rely on their hearing in certain situations, such as traffic. Yet, such safety standards must be maintained for impaired persons without the absolute necessity of an implant or even nudging them to decide for an implant, potentially leading to ethical conflicts. In implant care, safety refers to the feeling of security concerning implant functionality and therefore feeling safe in everyday life [18].

*Participation* can be seen as “a person’s involvement in activities that provide interaction with others in society or the community” (p. 2148) [36]. Certain norms and beliefs of a society/community regulate the mechanisms of (non)involvement of individuals. The understanding of disability, disease, and health, and the judgment about the need for treatment differ regarding different cultures, subcultures or religious beliefs [37]. Thus culture, defined as “The shared, overt and covert understandings that constitute conventions and practices, and the ideas, symbols, and concrete artifacts that sustain conventions and practices, and make them meaningful.” (p. 1610) [38] also affects the evaluation and acceptance of medical implants. The decision for an implant may affect participation e.g., by being seen as part of or no longer/ belonging to certain cultures or groups [18].

*(Social) justice* addresses the equitable distribution of benefits and burdens to individuals in social institutions, and how individuals’ rights are realized [39]. Inequalities in daily life conditions result from unequitable power, money and resource distribution and reason major parts of health inequities [40] undermining the human rights’ basis. In healthcare and implant care, this relates to questions of equal access to adequate treatment. Stigma associated with health conditions can negatively affect health-seeking behavior, engagement in care, or adherence to treatment [41]. Varieties of discrimination resulting from stigma can affect social acceptance of individuals or groups, reduce individual opportunities and fuel social inequalities [41].

A *sustainable* healthcare system “improves, maintains or restores health, while minimizing negative impacts on the environment and leveraging opportunities to restore and improve it, to the benefit of the health and well-being of current and future generations.” (p. IV) [42]. Implantation entails ongoing navigation of implant care and continuous (self)management of the disease and implant [18]. This demonstrates the importance of sustainable implant care, e.g. relating to therapy costs, long-term prognoses and continuous technological development.

### Aim

The scientific debate on ethical values in implant care is often determined by theoretical analyses. This is undoubtedly a meaningful contribution to implant ethics. However, the individual decision on implantation is made in a local context, influenced by immediate circumstances and challenges (e.g. access to healthcare services, personal preferences, cultural values, individual living conditions) [43]. The same applies to the impact of the implant on everyday life. Empirical data on implant-related ethical aspects from a patient’s perspective may help to better display the implant-specific circumstances and experiences with decision-making for or against an implantation and understand more about living with an implant. Examining the reflection of ethical principles in implant-wearers’ lifeworlds can provide valuable information for theoretical debates. A scoping review is a suitable form of knowledge synthesis, to map key concepts, evidence types, and research gaps by systematically searching, selecting and synthesizing existing knowledge [44]. Therefore, we conducted a synthesis of the existing evidence regarding ethically relevant psychosocial and cultural aspects in cochlear, glaucoma and cardiovascular implants in empirical patient-centered research.

## Methods

Our methodical approach is based on established methods and recommendations on conducting scoping reviews (e.g., PRISMA-ScR) [45–48]). A 5-phase scoping review framework was applied, including research question development, studies identification and selection, data collection, and result assembly [45, 46]).

Databases searched were: EBSCOhost, Philpapers, PsycNET (PsycArticles), Pubmed, Web of Science, and BELIT. Initially, we performed single, limited searches with search terms derived from core concepts (fundamental ethical values, patient experiences) and analyzed keywords and index terms of relevant hits. A suitable sup was developed testing Medical Subject Headings (MeSH terms), Boolean operators, truncations, and various search term combinations. In case a complex search algorithm was unfeasible for a database, we searched for implant-related articles in general (Philpapers, PsycNET (PsycArticles), BELIT). The final search algorithm for Pubmed is presented in supplementary material A. Literature searches were conducted on 25 March 2020 and updated on 28 April 2022. The final results were exported to a literature management program, duplicates were removed.

### Eligibility criteria

Eligibility criteria were developed iteratively during literature search and determined post hoc. Eligible studies were research articles in German or English, published since 2000. Regarding population, intervention, and outcomes, eligibility criteria are presented in Table 1.

**Table 1:** Eligibility criteria

	**Inclusion**	**Exclusion**
*Population*	(prospective) implant-wearers or their significant others (as long as thematic focus laid on impact of implant-related aspects on its wearers)	patients with specific comorbidities or underlying diseases, e.g., Usher’s syndrome
*Intervention*	Cochlear implants; glaucoma drainage or filtration implants (e.g., Ahmed valve implant, iStent); passive cardiovascular implants (valve replacement, coronary stent)	hearing loss, glaucoma or cardiovascular disease without any implant reference
*Outcome*	fundamental ethical values in relation to one of the three implant types; implant-related societal/cultural aspects (e.g., cultural identity, discrimination experiences, sense of connectedness or belonging to a certain social or cultural group, norms, expectations, acculturation, social acceptance, participation); psychosocial aspects (e.g., self-concept, self-confidence, personal identity, communication, social interaction, social relationships, personality, psychosocial functioning, psychological (not pathological), social well-being, lifestyle, self-esteem, employment, education, life planning	quality of life (QoL) outcomes, quantified with health-related or generic questionnaires

Qualitative studies were included if relevant outcomes were addressed in data collection or analysis, e.g., in interview guidelines or emerging themes. Quantitative studies were considered relevant if specific patient-report measures, addressing one of the above-mentioned aspects, were used. Studies based solely on generic questionnaires were excluded. Articles whose primary outcome(s) did not include the above-mentioned but presented relevant data regarding ethically relevant aspects of the respective implants were also included.

### Data selection and charting

The screening of articles against the eligibility criteria was performed in duplicate. In case of disagreement, a third person was involved. Further disagreements on the eligibility of articles were resolved by discussion among the three reviewers.

A descriptive-analytical method applying a common analytical framework to all research articles and collecting standard information of each article [45] was chosen. Independently developed data extraction forms were compared and integrated into a final template [46]. Initially, 10% of studies were charted in duplicate; results were compared to determine whether meaningful data extraction was allowed ensuring all relevant data was consistently captured across researchers. The remaining records were charted independently (C.H, M.L., S.S.). Data extraction was approached as an iterative process, so that the template was constantly edited, supplemented and refined in consultation within the team.

Extracted data included year of publication, country of publication, type of implant, research question/aim, population characteristics (e.g., age, gender, ethnicity, religion), methodological approach and resulting evidence regarding ethically relevant aspects as defined in the introduction. A list of all variables in the final data extraction form is presented in supplementary material B.

### Preparation, compilation and assembly of the results

We combined a descriptive numerical analysis with a thematically organized narrative synthesis of the findings. The descriptive numerical analysis was based on extracted data (e.g., publication year or methodology). Regarding the narrative synthesis, the introduced fundamental ethical values acted as conceptual supercategories according to which the included literature was thematically organized and structured. We derived topics for each article depending on applied questionnaires, surveys, single items, (sub)themes, (sub)categories, (sub)headings in the results section, interview topic guidelines or eligibility criteria (in the case of reviews). These were assigned to the ethical values. (The selection of the ethical values building the conceptual categories of this review was based on a preliminary literature research on ongoing theoretical and less empirical bioethical discussions on medical implants in general and these three specific implant types. We did not include the analysis in the current review in order to keep the focus on patients’ experiences and the existing empirical research.) The results of the included empirical studies were summarized in the narrative synthesis. Text passages regarding relevant keywords (e.g., self-confidence, discrimination) were also recorded and thematically organized accordingly. In the following, the results are presented along the previously reasoned *fundamental ethical values* (conceptual frame) and therein their thematic placement depending on the topics and keywords that occurred.

Considering the interconnectedness of ethical values, suggested classifications may give an overview of relevant concepts without striving for extensive theoretical comparisons of terminology.

## Results

**Fig. 1:**
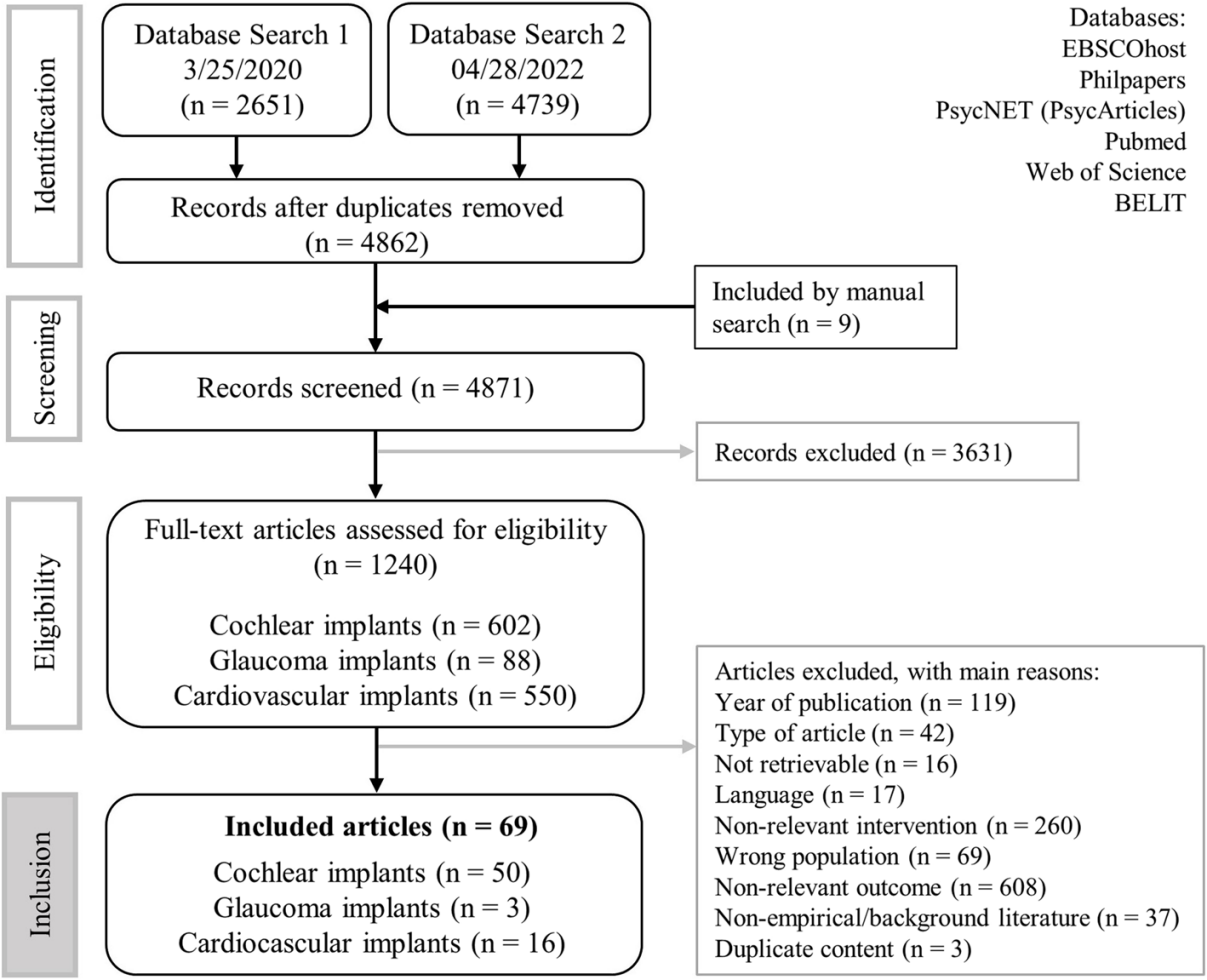
Flow chart of this review’s search strategy (CI-cochlear implants, GI-glaucoma implants, CVI-cardiovascular implants, n-number of records)

Electronic searches identified 7513 citations, resulting in 4862 unique citations to be screened for inclusion (flow chart, Fig. 1). In the screening process titles and abstracts of the articles were assessed, resulting in 1240 citations being retained. The full texts of these articles were assessed along the inclusion criteria. Through backward citation chaining, an additional 9 citations were identified, resulting in a total of 69 articles (CI = 50, GI = 3, CVI = 16).

### Numerical analysis

**Fig. 2:**
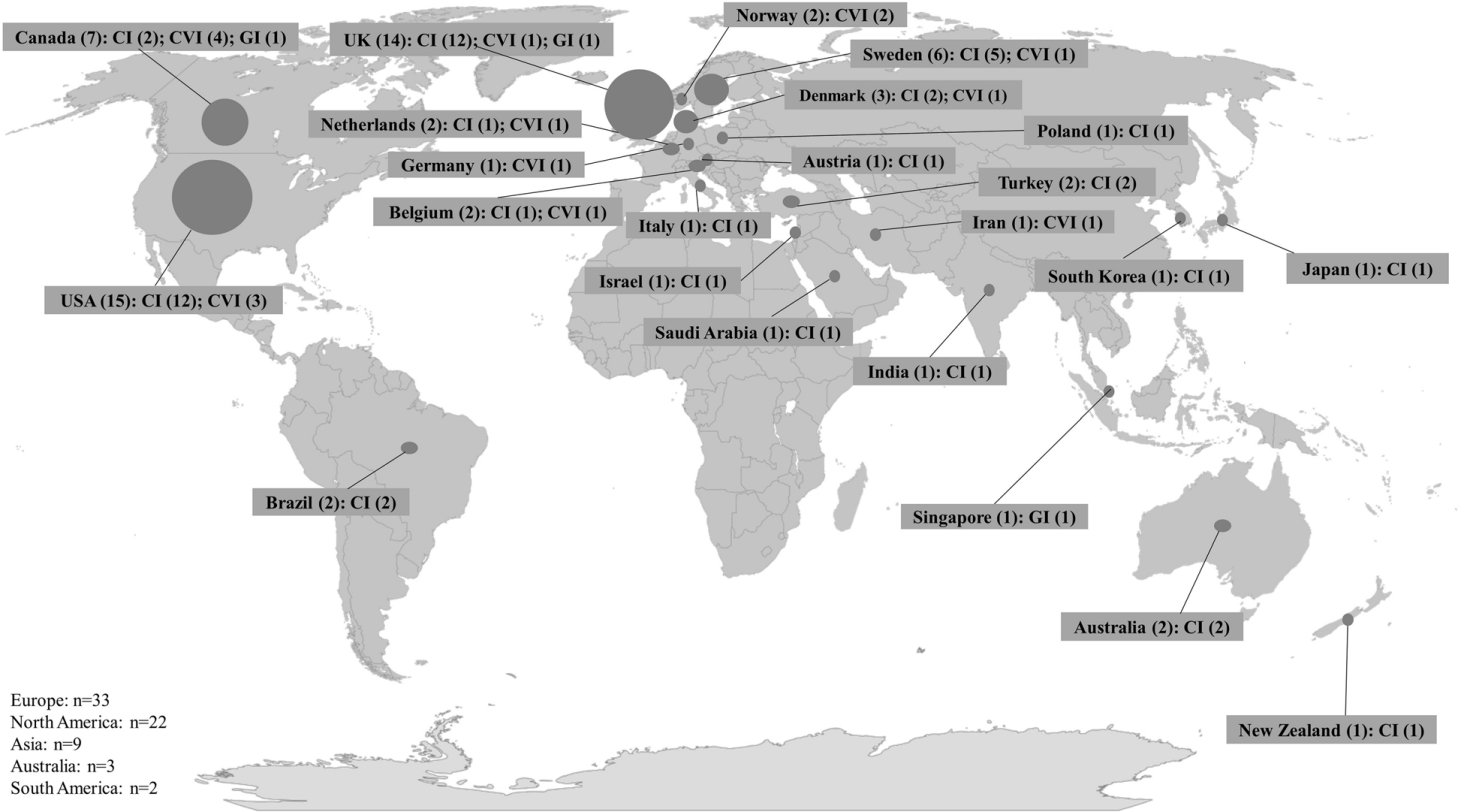
Number of articles per country and continent overall as well as in each implant field. Source: Map adapted from geographyteacherytc via Pixabay (in accordance with Pixabay Content License) (CI-cochlear implants, GI-glaucoma implants, CVI-cardiovascular implants)

Articles were published across North America, South America, Europe, Asia and Australia (see Fig. 2). Out of 50 articles addressing CI, most articles were published in the USA (*n* = 12) and UK (*n* = 12). The 3 articles addressing GI were published in Singapore, UK and Canada. With regards to CVI, out of 16 included articles, most were published in Canada (*n* = 4) and USA (*n* = 3). A detailed listing of all publications and the corresponding location is presented in supplementary table 1.

**Fig. 3:**
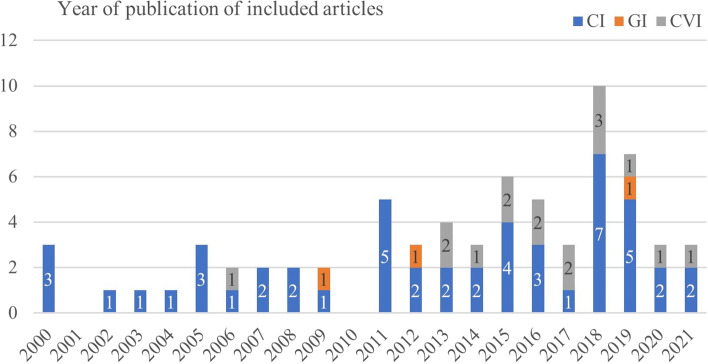
Year of publication of included articles in the respective implant fields (CI-cochlear implants, GI-glaucoma implants, CVI-cardiovascular implants)

All included studies were published between 2000 and 2021 (Fig. 3). Apart from one article, articles addressing CVI were published from 2013 while articles addressing CI were published nearly every year since 2000. In 34 studies a qualitative approach was chosen, mainly based on interview data (*n* = 32). Overall, 28 studies applied quantitative research methods, 6 used a mixed approach and 2 studies conducted reviews (Table 2). In the field of CI, qualitative methods (48%) were applied to a similar extent as quantitative methods (46%). Regarding GI, one article each applied a qualitative, quantitative or mixed design. With CVI, qualitative methods were applied in over half of the articles (53%), compared to 23% quantitative methods and 18% of studies applying a mixed approach.

**Table 2:** Methodical approach of included articles

	CI	GI	CVI	Total
Qualitative	24 (48%)	1 (33,3%)	9 (56%)	34
Quantitative	23 (46%)	1 (33,3%)	4 (25%)	28
Mixed	2 (4%)	1 (33,3%)	2 (13%)	5
Review	1 (2%)	-	1 (6%)	2
Total	50	3	16	69

Number of articles (percentage) applying qualitative, quantitative, mixed methodical designs or reviews in each implant field (CI-cochlear implants, GI-glaucoma implants, CVI-cardiovascular implants)

### Narrative synthesis

Based on the thematic sorting of relevant topics alongside the fundamental ethical values the main results of the included articles were summarized in the following narrative synthesis. An overview of the thematic sorting is presented in the supplementary table 2.

Some of the included studies refer to data from prospective implant-wearers, meaning individuals contemplating implantation who have not (yet) received an implant. These are included in the following description of 'implant-wearers'.

The vocabulary used in the narrative synthesis to address certain phenomena related to the implants mirrors the concepts and understandings in the original sources. Due to the fact that most of the empirical literature comes from medical and healthcare research, certain terms such as "disability" or "patient" in the narrative synthesis were adopted from the vocabulary used in the data source and contain no valuations made by the authors in this section.

#### Cochlear implants

Empirical evidence of ethically relevant aspects mainly related to informed, shared and parental decision-making (DM), post-implantation impact on autonomy (in device usage) and freedom of action, self-relation (body and technology; ability and disability), (Deaf) identity and participation in the hearing society. Concerning sustainability and privacy little to no data was available. In the narrative presentation of the results, no explicit overaching distinction is made between prelingual and postlingual hearing-impaired individuals and CI implantations; however, when refering to specific study results the respective population specifics are indicated clearly. Furthermore, this will be taken up in the discussion section.

With respect to autonomy, several studies assessed the perceived amount and quality of information and knowledge as a basis of *informed DM* and consent. Individuals reported on their level of satisfaction of CI-related health information and their experiences varied from feeling well-informed [49, 50] to poorly informed due to the-lack-of/or superficial information [51–53]. More in-depth information was desired [51, 54]. Satisfaction with the provided information may depends on which process step of implantation the implant recipient was [55] and opportunities for peer group exchange [49]. The following sources of information were perceived as reliable: media [56], lip reading class [51], other implant-wearers [54, 57, 58], peers [49–51, 53, 57–59], family/ friends [51, 56], internet [49, 51, 53, 57, 59], online forums [51] or Deaf organizations [53], health and implant professionals (e.g. implantation center, manufacturers) [49–53, 55, 57–59]. With regards to *shared decision-making (SDM)*, professional advice was perceived mostly positive [53, 59]. However, some deaf parents opting for CI for their deaf child perceived advice given by professionals to be one-sided and incomplete with an exclusive focus on the medical approach (implantation) and felt pressured to implant as early as possible [53]. In some cases, difficulties in processing information given by professionals arose due to the patient’s feeling overwhelmed and could manifest in trusting professionals out of ''blind faith'' (p.142) [60]. Regarding DM, most parents in one study (61%) felt they had no choice, stating to simply have complied with the referral decision [50]. The process of weighing among different opinions and information sources was perceived as important and needed to be supported by evidence-based information [58]. Although trying to inform themselves extensively, parents reported feeling overwhelmed before and shortly after implantation [57]. This ties in with *surrogate DM*; parents expressed concerns and difficulties about deciding for their children, who are not yet able to consent and make this decision in an autonomous way, wondering about how their children would evaluate this decision in the future [53, 57, 61].

The desire to live independently reportedly contributed to the decision to have a CI [55], assuming CI to play an important part in enabling more *autonomous, independent* and *self-determined lives*. This assumption was supported by other studies, e.g. depicting a post-implant transformation “from someone in need of help to an independent human being” (p. 543) [54]. CI-wearers felt less dependent on others [50, 52, 54] and had overall increased perceptions of independence [49, 50, 52, 62–65]. CI-wearers indicated feeling more informed (e.g. through increased sound perception [52], increasing knowledge and understanding of situations [64]), more assertive [66] and empowered through the implant [54, 64]. Furthermore, individuals’ perceptions of autonomy increased [54, 63–65] as well as their self-reliance [56, 57] and self-efficacy [54]. In terms of being emotionally autonomous from their parents, adolescents with CI demonstrated similar adaptive and maladaptive forms of emotional distancing from parents compared to hearing peers [67]. However, in another study parents worried the family’s overprotection (due to fear of implant damage) could increase the child’s dependence [64].

Autonomy was also relevant in *device usage*. For some CI-wearers a deeper understanding of the technology was lacking [68] potentially hindering self-determined usage of their implant. Similarly, the importance of acquiring skills in device management and getting used to the implant in daily life was illuminated in several articles [49, 52, 63, 64, 69, 70]. With increased ability in implant-management, CI-wearers could make use of the opportunity to turn their own hearing on and off [70, 71]. Against this stand feelings of dependence [68], anxiety or concerns about implant failure [49, 52, 57] as well as the impression of parents that their child is totally reliant on the implant [57].

In addition to increased autonomy and independence relating to freedom, the CI was associated with *freedom of action***.** Some CI-wearers indicated restrictions imposed by hearing loss to be lower post-implantation [64, 66] expanding the scope of possible actions. In this context, the implant was described as providing practicality and agility in life [64], enabling the CI-wearer to enjoy mundane activities [72]. The CI could bring about new or refined abilities in terms of hearing, e.g. using the phone, listening to music or playing an instrument [56, 64, 66, 69, 73–76]. (Re)gained hearing ability could in turn elicit independence which was associated with “the freedom to come and go, realize, think, act, be able to dream, and restart and rescue life projects” (p.6) [64]. However, in one study examining the perceived experience of hearing loss as a functional impairment, CI-wearers reported significantly higher levels of feeling limited by hearing loss compared to deaf individuals without CI [77].

The implant can be of a *restrictive* nature itself, necessitating the implant-wearer to refrain from certain activities in daily life due to the risk of damaging the implant [61, 63, 78]. Also, the risk of electrical shock led to parents prohibiting their implanted child from playing sports [61]. Another study mentioned that CIs were perceived as “bulky to carry around” (p. 404) hindering the child [50]. Some CI-wearers struggled with the responsibility of device-management [49]. Communication breakdowns, taking care of the device [76], the hardware and fragility of the device [49] (e.g., short battery runtime, high cost [54]) led to frustrations.

A CI affected identity in terms of the individual’s relation to themselves and their body, personality and cultural belonging. One article directly examined embodied identity development in connection with CI [17]. Regarding *self-relation,* the CI led to increased self-confidence [49, 50, 52, 56, 62, 64, 66, 72, 78] and feelings of self-worth, self-value and self-acceptance [52, 54, 64]. Comparing self-esteem of CI-wearers with other reference groups elicited heterogeneous results [79–81]. Also, one study indicated that some young people with CI struggled with self-concept issues [82], whereas another study did not find significant differences between adolescent CI-wearers and typical developing adolescents regarding self-concept scales [67]. With regards to sense of coherence, most of the examined children with CI had a strong sense of coherence (global tendency to view life situations as comprehensible, manageable and meaningful) [83]. Regarding *personality*, data from one study showed that two years post-implantation the CI had no effect on any of the Big Five personality factors [84]. However, in another study some parents attributed development of confidence and a more outgoing personality to the CI [72].

The implant was valued differently in *relation to the body*: it was seen as a natural part of the individual’s life [71]; or perceived to be embarrassing [50]. Some CI-wearers were self-conscious about the appearance of their CI equipment [50, 72, 76, 78] and/or tried to hide the device [49, 78, 82]. The CI was perceived to be a visual sign distinguishing implant-wearers from hearing people [78]. Here, the age at implantation could be a decisive factor in individual’s relation to their body, since there is evidence that an early age CI-implantation related to higher levels of physical self-concept and perceptions of attractiveness [67]. The CI can have a *transformative and existential* impact, revolutionizing the implant-wearer’s life [64]. Transforming CI-wearer’s body the implantation was seen as a disruption of the unity between body and self-perception (“being shaken” and “losing one’s own foundation”) [70]; this transformation (“significant revelation as well as the emotionally loaded starting point for the subjects coming back to life” (p. 119) [52]) led the CI-wearer to feel the world in a radically different way [70].

Having a CI can also impact (self)-perceptions with regards to *disability and Deaf identity* [70]. In this review Deaf identity is understood as belonging to the Deaf culture based on shared experiences with deafness, common language and communication mode and shared understandings on Deafness as cultural variety, while ‘being deaf’ refers to the health condition in terms of hearing capabilities. Local cultural beliefs on disability (e.g., an equation between deafness and stupidity) may lead to an internalised shame [17]. The analysed literature did not provide a clear picture on the impact of CI on individual’s (self-)perceptions on disability [66]: the CI could bring about (1) a new sense of disability [70] for the implant-wearer, realizing how much has been missing pre-implantation [50, 70] or realising that hearing people can simply hear effortlessly [17]; (2) a feeling of being more deaf and dependant after sequential CI [78]; (3) a feeling of “fixing” the disability of deafness through CI [78].

Similarly, variability in the participants’ accounts with regards to cultural identity was depicted [49, 55, 56, 68, 71, 78, 85]. Some CI-wearers felt more connected to the hearing world [49, 56, 85]; others identified themselves as being both hearing and deaf [49, 71, 78], acknowledging their deafness but identifying with their ability to hear via CI [78]. Some participants not using sign language reported not feeling fully integrated into the hearing or the Deaf world [56]. Even identifying themselves as deaf, participants rarely aligned themselves with being culturally Deaf [49, 55, 78]. No conclusive evidence was found on the impact of the (non)use of CI with respect to the self-perceived identity as ‘hearing’ or ‘Deaf’ among deaf and hard-or hearing individuals. However, it is indicated that CI-wearers tend to bicultural or hearing identity, whereas deaf individuals without CIs showed more Deaf acculturation [77, 86–90]. There is also evidence of educational setting (mainstream, special or mixed schools) impacting identity formation [80, 86]: deaf adolescents attending mainstream educational settings had significantly lower scores in Deaf acculturation and higher hearing acculturation [80]. Adolescents who did not maintain a stable educational setting were not able to establish a cultural identity [86].

The CI can impact individual’s safety in everyday life: with the CI the wearer is able to perceive environmental sounds or warning signals [50, 62, 64, 71, 91], keeping them safe e.g. from approaching cars in traffic [66]. In the same vein, parents felt free to let their child with CI play outside since they would be aware of the environmental sounds [57]. The nightly deafness after removing the CI however led to feeling insecure (e.g. risk of missing alarms) [50]. Moreover, concerns about functioning and the effect of having an implant inside one’s head were expressed, wondering “whether it’s going wrong on the inside” (p. 38) [68].

Regarding privacy, CI can bring to its wearer a feeling of empowerment that enables the implant-wearer to do things alone, in private [64]. the visibility of the implant, however, discloses private information on the individual’s health condition. Not wanting to be judged as “deaf” or “not fitting in” with the environment, implant-wearers hid their CI [49, 78, 82], wishing it could be smaller, less noticeable [49].

Considering that (non-)hearing fundamentally affects the ability to communicate and relate to others [70] the CI influences participation. Several included articles, including a scoping review [65], indicated a positive influence of the CI on *participation and social life* [52, 57, 65, 76, 78, 92]. Post-implantation, CI-wearers regained their place in the hearing world as equal communication partners [49], feeling a greater sense of connectedness [52, 54, 56]. Confidence in interacting socially was increased post-implant [50, 52, 54, 56, 62, 64, 66, 71, 74]. CI-wearers felt encouraged to be initiating and light-hearted in social interactions [54, 64] and perceived their social environment as more proactive in starting conversations post-implantation [54]. Having a CI was described as “after years of being a mediocre person, you become someone who other people recognize and reckon with” (p. 118) [52]. Post-implantation, the nature of interaction changed [93] and new dynamics in personal relationships were established [54, 74]. Social support was generally important for CI-wearers [94] facilitating participation and inclusion [72].

The CI in relation to *education or employment* was addressed by several studies. While deaf parents contemplating a CI for their deaf child were divided regarding the importance of their child’s participation in mainstream (hearing) education in creating future opportunities in education and employment [53], CIs were often considered important and beneficial for education or work [50, 54, 57, 62, 65, 66, 68, 78], e.g. helping to better understand lessons [68] or being “able to perform work tasks just like anyone else” (p. 543) [54]. Also, the educational setting impacted on friendship patterns [72, 83, 95]: children in mainstream schools had larger and closer social networks compared to children in special schools [83]. Some barriers and challenges still occurred post-implantation [65, 75, 77, 95] (e.g., cultural differences with hearing people [77], or struggles to hear and follow conversations in groups or loud environments [72, 78, 95]), leading to “social deafness” (p. 481) [72]. Difficulties also related to lacking awareness for some of the subtleties involved in peer interactions indicating a need to improve social skills [72]. Moreover, exclusion from social activities or sports due to the implant led to frustrations [78].

*Belonging to the Deaf or the hearing cultures* was also important for participation, potentially being influenced by social norms and convictions on normality [60, 63]. This was a possible reason why parents might have attached different levels of importance of child’s participation in the hearing world [53, 96]. Participants without CI were more likely to report socializing with deaf friends and participating in Deaf culture activities whereas those with CI were more likely to socialize with hearing friends [77]. Similarly, hearing acculturation (being significantly higher in adolescents with CI than without) was positively related to socialization with and acceptance by hearing peers and negatively related to socialization with and acceptance by deaf peers [80]. In contrast, marginal Deaf identity (feeling alienated from both the Deaf and hearing communities) was associated with difficulties in peer relationships [89]. Perceiving themselves as dissimilar from hearing peers was negatively associated with the CI-wearer’s well-being [85]; this finding may be also related to results of another study, indicating that individuals with a hearing identity had significantly greater feelings of limitation than those with a deaf or bicultural identity [77].

Against the background of hearing as a social norm, the visibility of the implant can elicit worries of *acceptance by others*, e.g., concerns of looking different “’like an alien’ […] with ‘metal bits on my skull’” (p. 259) [70]. The fear of alienation prior to the implantation decreased after the implantation which was seen as a transformation from a state of alienation to normality—as not being seen as different from everyone else, the “hearing people” [54]. Being able to function more like a hearing person resulted in feeling less different [49]. The integration of the device into the CI-wearer’s daily lives at an early age may support acceptance by others and increase participation [76]. A study demonstrated that a vast majority of parents (88%) experienced that their child with CI is easily accepted by other children in the classroom; nevertheless, 12% still reported experiences of social isolation of their child [97]. The extent to which CI-wearers perceived others to be bothered by their hearing difficulties also strongly influenced psychological well-being [94].

In terms of justice, difficulties in accessing adequate healthcare services were addressed in several studies [50, 54, 55, 61, 63, 69, 91, 93]. Travelling to the CI-center was an obstacle for CI-wearers, requiring support from their social environment or health services [55]. Other burdens included personal expenses for equipment [54], rehabilitation [63] or out-of-pocket contributions to implantation/device cost [69] and device updates [61]. Some patients experienced institutional discrimination based on ethnicity or race hindering access to CI services [91, 93]. Discrimination related to hearing loss was also addressed [55, 63, 64, 78, 87]. Negative prejudice of others towards deafness based on the perception of deaf people as being “dumb” impacted individual’s decision opting for CI as protection against prejudice and possible bullying [78]; the stigma of disability could be reduced by using CI as a new, expensive and high-tech device [64]. A comparison between the perception of stigmatization of deaf students with or without CIs showed no difference [87]. On occasion, the fear of prejudice and social discrimination remained for parents also after implantation [63].

Sustainability related to future technological and scientific advancements in contrast to the irreversibility of implantation [55] and the availability of long-term implant related healthcare: Uncertainty regarding healthcare policy, e.g., in case of equipment problems or loss, can intensify worries about losing or damaging device equipment [52]. Still, individuals felt confident regarding advancements technical science [52].

#### Glaucoma implants 

A prominent topic concerning GI was SDM. No relevant information on identity, privacy or sustainability was available. Compared to CI and CVI very little information addressing GI was found.

Relevant information was depicted regarding the realization of autonomy in SDM, whereby the *patient-doctor relationship* (PDR*)* played an important role [98, 99]. A trusting relationship was essential in developing comfort when deciding to undergo GI surgery. Trust in one’s own healthcare providers and the perception of a SDM-process influenced DM positively [98, 99]. This was i.e. attributed to glaucoma as a disease being associated with uncertainty due to its “unknown nature and symptoms” [98]. One study referred to the surgeon’s ethnic-specific expertise as a valuable factor contributing to the patient's well-being [99]. Autonomy in DM on GI was strongly impacted by the feared loss of eyesight, whereby surgery was perceived to be the *only option* to avoid blindness. Eyesight being perceived to be essential for maintaining individuality and independence, reinforced the perception of inevitability and necessity of the procedure [98]. The decision was also partly determined by the gradual severity of the disease. Glaucoma patients willing to accept an implant were mainly (appr. 63%) those who had more severe glaucoma [100].

Freedom was mostly related to restrictions in everyday life resulting from continuous eye drop therapy. Although patients reported that their eye drop prescriptions were generally simple and effective [98], lack of adherence and medicinal treatment was generally problematic [98, 100]. Compliance issues and other frustrations with pharmacotherapy (e.g., the eye drop regimen determining their everyday life) were factors that reinforced the decision to undergo surgery [98]. Participants viewed the procedure as an opportunity to maintain their current level of activity and QoL [98].

Relating to safety, patients reported that the largest impact of glaucoma was emotional distress and fear of potentially losing eyesight [98]. In the same study, a participant expressed that being able to maintain vision through surgeries meant that they “can be safe” (p. 34) [98].

In terms of participation, glaucoma felt like an invisible condition which families and coworkers were not aware of [98]. Disruptions and frustrations evoked by the condition could therefore be overlooked and ignored by others [98]. The main factor in the extracted data which refers to participation is again the fear of blindness, since vision was perceived to be essential for participation: “to engage in the world”, “to live a life with meaning” (p. 31) [98].

In terms of justice, only one study from Canada reported that the potential cost associated with glaucoma treatments, including eye drops caused frustration among some patients [98].

#### Cardiovascular implants 

Out of 16 articles, 15 articles dealt with valve replacements and one article addressed coronary stents. Autonomy in DM, SDM, freedom and feeling safe against the background of facing mortality were prominent topics. No relevant information was available concerning sustainability.

Autonomy was mainly related to DM. One study directly addressed autonomous DM [101]: it was seen as a balancing act of dependence on physicians and autonomous appraisal capacity, where “autonomous trust” could be established through positive encounters with physicians [101]. Trust in the physician established a feeling of confidence regarding DM [102]. Patients felt that the decision was shared between patient and physician [103], generally feeling included in DM [104]. But other patients also reported that the decision was more likely to be made by the physician [103]. With regards to the final decision on valve choice, most patients specified that the decision was made by the physician; with the patient’s consent [105]. Health professionals were valued in the DM process as co- ‘deciders’ who take part in the decision or even take it off of the patient [103, 106, 107]. Depending on personal identity and situational circumstances valuing of autonomous DM varied [101, 106]. Whereas for some participants it was important to make the decision on their own [101, 103], the wish to be relieved of the decision, following what experts recommend due to feeling completely overwhelmed [106] was also expressed.

Several studies addressed issues related to *informed DM* (e.g., availability, sources and needs of information) [101, 103, 105–114]. Patients with mechanical CVI were better informed than patients with biological valve replacement [108], while coronary stent as intervention was overall not well-known [109]. Especially in emergencies, where patients could not be thoroughly consulted, more implant- and disease-related information were desired post-implantation [109]. Sources of information were professionals [103, 107, 114], hospital ratings [110], digital information platforms [105], rehabilitation programs [112]. Pre-implantation consultation also was a factor in DM [101, 108, 114]. Some patients preferred an “honest approach” (p.434) while for others the information communicated in consultations evoked negative feelings, as patients were confronted with their own mortality; some felt that the information focused on the risks rather than benefits [114]. Two studies reported mostly positive attitudes regarding information sharing and consultation by and with physicians [101, 103]. Variability in patient preferences and values was stated to highlight the importance of well-founded and informed SDM [115].

Furthermore, the *availability of options* is essential in the realization of self-determination. However, in some cases implantation was seen as the only option [101, 106]. Given the patients’ health status, some “felt that they had little choice” (p. 437) regarding the type of procedure having undergone several open-heart surgeries already [114], leaving TAVI to be the only alternative.

Heart disease can severely limit patient’s freedom by imposing physical restrictions. Symptoms such as breathlessness or fatigue aggravate engaging in physical activities and lead to functional restrictions in activities of daily living [102, 107, 114] limiting the opportunity to live a full life [114]. Some patients were dependent on medications to manage their symptoms [102]. Post-implantation, reduced symptom burden [104, 113, 114] enabled patients to stay independent and plan more confidently for the future [104]. The wish to gain more freedom is mirrored in the patient’s valuing a quicker time to return to independence associated with TAVI compared to SAVR [116]. Also, the implantation could facilitate the treatment of other health conditions [114]. Nevertheless, living with complex health challenges, some patients still struggled with limiting symptoms post-implantation [104, 111–114].

The CVI itself could bring about *limitations in daily life* of implant-wearers: continuous anticoagulation medication and possible side effects [106, 113], frequent blood testing and adjustment of drug usage [106, 113], lifestyles changes (e.g., refraining from contact sports or shaving with a blade, diet modifications [106]). A study examining valve-specific interferences with QoL showed that 23% of participants stated being afraid of eventual bleeding complications due to anticoagulant medication, 19% perceived regular blood samplings and medical visits to be disturbing; 24% stated feeling disturbed by the valve noise (mechanical valve) [105].

Regarding identity and *self-relation*, patients felt worthless and burdensome due to their loss of independence caused by physically limiting disease factors. In this context, the implantation was positively depicted as having *a life-changing impact* [114], experiencing a “new start in life”, “returning to life” post-implantation (p. 154) [104]. One participant perceived a change in *personality*, from being nervous, aggressive and irritable before to a calmer and more patient character post-surgery [109]. The implant furthermore affected the implant-wearer’s *relation to their body*. Post-implantation, patients became very aware of their body paying more attention to signals and sensations [112]. Such increased bodily attention, e.g. focusing on the surgery scar, could entail a psychological burden, which needed to be compensated with (1) the development of a fit body [112] or (2) hiding the scar [112, 113]. On a similar note, after stenting, some participants gained the sense of having physical impairment, feeling “like a broken dish” (p. 184) [109].

A topic specific to CVI relating to safety was the health threat imposed by the heart disease (progression) in the form of *impending death* instigating patients to face their own mortality [104, 112, 114], potentially shattering their feeling of safety. In this regard, a sense of *personal safety* was rebuilt by the implant [114]. Also, participating in rehabilitation programs and receiving information and training instructions contributed to feeling safer [112, 113]. In some cases however, a sense of insecurity remained post-implantation with patients worrying about inadequate follow-up treatment, valve sound, anticoagulation side effects or complications [113]. Another article reported on the anxiety and fear for the future induced by insecurity regarding the implant functioning (possibility of clogging in stents) and not knowing about the stent lifetime [109].

Regarding privacy, the closing sound of a mechanical valve which may be noticeable to others caused stressful situations for the patient, who tried to conceal the sound of the valve through talking or moving, layers of clothing or installing a wall clock with a sound similar to the valve [113].

Physical limitations associated with cardiovascular diseases can lead to social restriction [104, 114], illuminating the relevance of CVI for participation. Furthermore, feeling responsible to maintain the best possible health to participate in day-to-day-activities [107], also as an obligation to their relatives [101], motivated patients to seek or accept treatment. However, in some cases TAVI did not dissolve social restrictions with patients being old and afflicted by other diseases impairing the potential for a good social life [104]. Similarly, functioning problems were associated with concerns of being socially accepted post-implantation [109]. Informing family and friends of an upcoming open-heart surgery was depicted to be emotionally draining [106].

Relevant to justice, logistical and economical barriers and facilitators in accessing healthcare services were addressed [102, 107, 109, 111]. Patients living far from the procedure site were concerned about travel burden [107], reported greater difficulty in assessing the procedure [102] and felt the need to have systems in place to assist with transportation [111] or social networks [107]. Patients were concerned about personal costs involved for travel [102, 107] and expressed concerns regarding financials and economic problems [109].

An overview of main results of this synthesis is presented in supplementary table 3.

## Discussion

In this scoping review empirical research into ethically relevant cultural and psychosocial aspects of CI, GI and CVI was examined and summarized to deepen the understanding of how fundamental ethical values can enrich or impact an implant-wearer’s life. Aspects related to the implant could be found in connection with all fundamental values addressed in this review, although they were not equally addressed in all implant areas. It has to be acknowledged that the types of implants and their applications differ and are therefore challenging to discuss on a meta level. Some ethical values and concepts are therefore more or less relevant regarding each type of implant and are discussed in the following.

### Autonomy

Patient autonomy is widely discussed in the medical and bioethics literature as a fundamental ethical principle in healthcare alongside understanding risks and benefits as a basis for informed DM and its implications for the physician’s role [25, 117–123]. These aspects are also underlined by the findings of this review, which reveal several weak points in terms of implementing patient autonomy in implant care. Perceptions of lacking or insufficient information, one-sided or biased professionals’ advice, pressure to decide for an implant or lacking disease awareness were depicted in patients’ perspectives and lived experiences as factors hindering autonomy. Empirical research on patients’ perspectives and lived experiences offers valuable insights regarding designing and providing transparent and comprehensible implant-related information.

The ethical literature also thematizes industry-sponsored research and physicians’ conflicts of interest in all implant areas. The existence of financial incentives regarding specific implant brands impacts prescriptions or maintenance, potentially undermining patient autonomy [120, 124–126]. However, this was not addressed in the empirical studies included in this review. Here, disclosure obligations and transparency on the part of the professionals could be valuable measures for patients to be in the know; this in turn requires patients’ ability to engage with and process the technological information on the different brands and choose accordingly—a matter that is worthy of further empirical research.

Furthermore, whenever the question arises as to whether patients are sufficiently capable of making decisions and consenting autonomously, e.g. in case of psychiatric patients [119], surrogate DM is a relevant ethical issue. Surrogate DM, “best interest” decisions and the child’s right to an open future are prominently discussed in the case of pediatric CI [8, 127–129], what distinguishes strongly the prelingual CI implantation of children from postlingual implantation in general. Accounts on parental challenges when deciding on CI for their child were also addressed in this review revealing parents’ distress and uncertainty. Further empirical research on surrogate DM may support the development of interventions to reduce parental stress in the course of DM, offer advice how to address the DM in conversations with their child in the future or support their child in identity building.

In view of the option of self-adjusting in electronic implantable devices it is questioned from an ethical standpoint whether or to what extent patients should have control over their devices [130]. The importance of assessing the patient’s or surrogate’s ability to remain responsible for the device is emphasized [119]. The analysis of the empirical research included in this review demonstrates that some implant users have difficulties to use the implanted device responsibly and autonomously, revealing the practical need to enable implant-wearers to autonomously manage their device in everyday life including an understanding of the device technology. Here, health literacy (HL) could prove to be a beneficial factor for device management. *Technology-related HL* [131], as a ‘dimension upgrade’ on established models of HL and specification of digital HL, could serve as a contemporary add-on to this concept [18].

### Freedom

On the one hand the implant can extend the implant-wearer’s freedom of action and autonomy, on the other hand, they depend on the proper functioning of the implant implying a loss of autonomy in case of technical implant failure. The technical dependency might also be intensified by concurrent psychological dependency [132]. This dimension of freedom related to implants is discussed predominantly in the context of neuroprostheses or neural implants [132, 133]. Looking at the findings of this review, these issues can also apply to a certain degree to non-electric implants such as CVI or GI. Perceptions of increased independence and the implant’s impact in lessening (or preventing impeding) limitations of the health condition were illustrated in the empirical literature in all implant areas. However, the implant itself was also reported to impose restrictions on the implant-wearer’s life. Furthermore, our results on CVIs indicate that the actual impact of the implant was difficult to assess or differed from expectations since comorbidities remained after implantation. Tying in with informed DM, this illustrates the importance of ensuring that patients have realistic expectations (e.g., regarding medication regiment, valve noise, post-implant comorbidities) implying corresponding demands on health professionals in consultation situations.

### Identity

Other than the intended reversal of personality changes caused by a disease [133] the possibility and moral implications of implant-related personality or identity remain contested. This discussion is predominantly conducted in the context of neural implants or brain-machine-interfaces (BMIs) [31, 119, 134–136], alongside debates on agency or free will [135, 137, 138]. On a more practical note, the possibility of identity changes elicited by medical interventions raises questions of appropriately obtaining informed consent [30]. When deciding to undergo the implantation, individuals may understand the possibility of identity changes, but cannot reasonably understand what *feeling like a different person* entails; highlighting the possibility of identity changes in discussions regarding the suitability of the implant for the individual is therefore essential [119]. However, the extent to which individuals were aware of the possibility of changes in identity or self-relation was not assessed in the empirical studies in this review. Further research on patients’ experiences could illuminate ways of integrating the issue of identity in implant-related counselling. With regards to cultural identity, ethical debates [139–142] are embedded in contemplations on disability identity, the definition of “normal” bodies, also questioning the nature and significance of (minority) cultures [7, 128], and touch on participation. In the case of CI, this applies especially to prelingually deaf children and their hearing or deaf parents who decide for or against a CI. Considering how Deaf people and hearing children of Deaf adults inhabit various in-between spaces, highlights the fluidity of cultural identities, deconstructing the notion of essentialist Deaf and hearing identities [140]. The various dimensions and intersections of implant-wearers’ cultural identity depicted in this review align with the concept of a fluid and complex cultural identity. Research efforts on cultural identity need to expand beyond CIs also to GIs and CVIs. The meaning of the implant in relation to the body and its impact on identity are discussed relating to issues of embodying technology like cyborgs, fusion of body and machine, predominantly in the context of neural implants, neuroprostheses or BMI [135, 138, 139, 143, 144]. On a more individual level, the transition that occurs in learning to hear with a CI is contemplated as a “total disruption” and “complete re-worlding” (p. 307) [143]. The evidence of the implant’s transformational impact on implant-wearers’ lives and its effect on self-relation summarized in this review supports this, not only regarding CIs but also CVIs. Our results furthermore suggest difficulties of adjusting body image regarding the visibility of implant components or implant-related scars and the perception of living in a scarred or disturbed body. Still, more insight regarding the meaning of implants in relation to body and identity is needed, further exploring implant-wearers’ experiences. 

### Safety

The research landscape of implants is dominated by issues of patient safety in the sense of medical safety, efficacy and legal regulations [145–147]. Furthermore, the ethical duty of the physician in case safety concerns arise towards already implanted types of devices is elaborated [126]. Issues of remote accessibility or cybersecurity are addressed, also relating to privacy [119, 120, 125, 148]. The awareness of implant-wearers of device-related cybersecurity issues, however, was not addressed in the literature examined in this review. Our findings surround other notions of safety, namely the establishment of safer living conditions through the implant and fears of implant failure. In view of the contribution of an implant to a safer living environment, implant-wearers’ lived experiences illuminate the ethical relevance of implants in terms of safety, going beyond the often-elaborated medical efficacy or safety risks. Further empirical research on the relevance of cybersecurity in everyday life of CI-wearers is recommended.

### Privacy

In general, experiences of privacy invasion or gained privacy are rarely covered in the research landscape, which focusses on privacy in the context of data protection of health information [149, 150] or security and privacy issues of implantable medical devices [132, 151, 152]. This threat to data privacy can be transferred to other implant (technology) that can assess and store health-related data (e.g., CI). This ethical issue gets particularly controversial with respect to a medical-technical progress along with digital data-dominated markets [153, 154]. With personal data being a digital currency in nowadays economy, the need for strict protection of personal (heath) data amplifies. Since this was not addressed in the empirical research included in this review, the extent of implant-wearers’ awareness on these issues remains unclear. A privacy-relevant subject that was addressed in this review, however, is the visibility (CI) or acoustic notability (CVI) of implants, disclosing private information regarding the health condition. With prejudices and stigma being central issues in health-related discourses, these aspects need to be considered as contributing factors to the role of privacy in implant care.

### Participation

Against the background of social norms of a hearing, seeing and functioning body, implants can strongly influence participation. One participation-related ethical issue addressed in this review regarding CIs is potential discrimination of non-hearing community members by valuing functional hearing as a defining cornerstone for ‘good life’ and thereby disregarding the facets of a pluralistic society [126]. Emphasizing implantation as a gold standard concerning social “functioning” and inclusion inevitably indicates the potential of social exclusion in implant care. In the example of persons with hearing loss, medical efforts mostly direct to aural communication only, disintegrating persons without implants. This hinders alternative inclusion efforts like teaching and learning/wanting to learn sign language [155], thereby further consolidating the normativity of conventional medicine. Furthermore, our findings showed that whereas CI and CVI facilitated social participation in the sense of removing barriers such as physical symptoms or communication difficulties, maintaining eyesight by means of GIs was perceived to be essential to engage in the world. Here, GIs present an opportunity to prevent visual impairment, which otherwise can jeopardize participation [156–158]. Aligning with our findings, CVIs enable implant-wearers to fulfill social roles and feel like a useful part of the community [18]. Social participation has been studied both empirically and theoretically in CIs; in CVIs and GIs however, there is a lack of research in this respect.

### Justice

In terms of justice, patient experiences concerning facilitators and barriers in accessing healthcare were addressed in this review. Cost and expenses were pointed out across all three implant areas. Travel burden or institutional discrimination were also factors jeopardizing equitable access. This aligns with research showing racial/ethnic and insurance disparities in pediatric cochlear implantation [159]. Health disparities are also discussed regarding GI among people of African descent [99]; Moreover, prescription of eye drops considering cost–benefit ratios was subject to geopolitical disparities [160]. Geographical injustice in medical device distribution or healthcare infrastructure implies an unequal access for respective ethnicities. There are endeavors to reuse medical implants to cover care demands in low- or middle-income countries; however, they are accompanied by ethical issues, e.g., when it comes to inequalities in state of the art of technologies. An already used device that is *re*used may not meet care standards in wealthier countries but is supposed to be ‘good enough’ for developing countries [161]. In this review, ethical issues are revealed that are emphasized by previous research. We strongly encourage further research in this regard including possible cultural differences in perception on, assessment of and attitudes towards implantable technology.

### Sustainability

Sustainability in implant care relates to (1) long-term treatment success (avoidance of reimplantation) entailing continuous access to healthcare services or implementing implant-related technological innovations, as well as (2) sustainable manufacturing of medical implants (e.g., ecological footprint, durability of implants). Sustainability is especially important for pediatric implantation, as these individuals often need to be able to sustain their CI for the rest of their lives, being dependent on long-term access as well as affordability of care. These considerations were reflected in the empirical research on CIs included in the review. At the system level, there were uncertainties regarding the long-term supply of technological equipment. Ultimately, however, confidence was placed in technological development. Continuous access to healthcare is intertwined with justice and also touches upon signclinical and financial sustainability of implant programs [162]. Environmental sustainability of implants is highly debated within the field of organ tissue and biomaterial implants [163, 164] and technological advancements and developments offer the opportunity to increase the sustainability of implants in various fields [165–169]. Such advancements reveal the need to reflect upon ethical questions of progress in technological implant development, e.g. how to proceed when higher performing implants indicate the replacement of an older device [125]. Patients’ perspectives regarding ecological considerations were not addressed in the included articles of this review. However, assessing and integrating patient perspectives in implant development might allow for insights in how to sustainably implement innovative implant technologies.

### General

The use, definition and operationalization of ethical values and terms in the reviewed research were often vague and unclear. Terms like independence, autonomy or freedom were often not clearly differentiated, at times aggravating the distinct relation of extracted data to an ethical value. This might be due to a missing orientation framework for research on ethical values in the context of lived experiences, conceptual difficulties, and limitations in the ethics expertise of researchers in this field. Furthermore, the amount of literature found in the respective implant areas differed significantly. Articles referring to CI accounted for a vast majority of results whereas hardly any relevant articles regarding ethically relevant cultural and psychosocial aspects of GI could be found. Also, the amount of relevant empirical literature addressing passive CVI was lower than for CI.

This imbalance may root in: (1) the visibility and noticeability of the implant, whereby CIs are much more visible than GI and CVI; (2) range of treatment alternatives, whereby CI and GI are possible but not ultimate treatment options, unlike CVI which can be lifesaving; (3) aspects of pediatric CI treatment such as surrogate DM, implant-related impact on functional and social development; (4) the cultural meaning and complexity of CI.

### Implications

#### Implications for individual care and healthcare programs

The entire care process surrounding implants should be considered, from (1) decision-making to (2) implantation to (3) subsequent rehabilitation as well as (4) long-term maintenance. Ethical aspects must be operationalized individually and systemically. This may include, e.g., psychological care, culturally sensitive counselling, and technological training and follow-up care. Such an approach prerequisites that research is increasingly integrated into everyday care. It is moreover required that implant-recipients, their significant others, professionals from different occupational fields, and organized groups, e.g., self-help groups, are sensitized and educated on ethical issues. Professionals are encouraged to reflect on their own ethical values and internalized norms, especially regarding bodily functioning, disability and physical integrity, and to what extent these may affect their recommendations, counselling or the course of treatment. More guidelines, interventions or trainings (e.g. eLearning-modules on the subject of implant ethics [170]), should be developed for both affected persons and professionals to address these issues and to provide guidance on how to deal with the individual situation in care and everyday life.

Regarding the technology we suggest implementing an *ethics-by-design* approach [171]. This approach considers ethical values throughout the entire process of technology development and deployment. The aim of *ethics-by-design* is to acknowledge ethical implications and eliminate or reduce potential problems already when designing a product. Adequate training for implant engineers as well as interdisciplinary teams including ethical expertise is recommended.

#### Implications for future research

Ethical aspects explicitly named as such have not yet been investigated extensively in the field of implant care. Although the field of CIs has been by far the most researched in this respect, all three implant areas are underrepresented regarding empirical research focusing on patient perspectives relating to ethical values; often-used *generic* measurements may not adequately reflect the individual lifeworld of an implant-wearer, potentially being unsuitable for in-depth research of ethical issues from a patient viewpoint. Alternatively, qualitative methods (e.g., group discussions, observations of counselling situations, diary studies) can help to capture first-hand experiences and perspectives of implant-wearers regarding ethical values.

Furthermore, there is a need for research with respect to *awareness of ethical relevance* regarding different influential factors in implant care, e.g., systemic circumstances, that may promote or hinder equity of access to implant care or ongoing maintenance. Research on these issues is also embedded in an overarching discourse on ableism, including normative convictions regarding bodily function, reinforced by increasing technological opportunities. Again, incorporating a patient’s point of view by specifically directed qualitative and participatory research can improve understanding of systemic issues in this context. There should be more empirical research in the field of patient-centered implant care, specifically investigating ethical dimensions, values and principles that are connected to patients’ concerns.

In total, these prospects can contribute to a successive uncovering and mapping of ethical aspects in implant care with the aim of integrating them in implant development and care guidelines.

### Strengths and limitations

Both data selection and data extraction were supervised and cross-checked by at least three researchers, in duplicate, and independently, allowing for internal validation of the proceedings. In addition, the search algorithm was gradually tested and refined and ultimately applied to several databases, resulting in a high degree of saturation of the relevant literature. Not limiting the search to a certain empirical study design enabled the coverage of a variety of research in this review. The extensiveness and actuality of the findings was maintained and enhanced by an update search. To the best of our knowledge, this is the first review of ethically relevant psychosocial and cultural aspects of CI, GI and CVI.

Considering the lack of an ethical framework and consensus of definitions regarding ethical principles and values in empirical research, it is conceivable that, although the search algorithm was very comprehensive, relevant articles not applying the exact ethical keywords used in this review were not captured. Since the included studies had hardly any explicit definitions or explanations of ethical principles and values, a *minimal level of interpretation* was required, potentially leading to an inadequate recognition of some text passages. To counteract this potential bias, careful data extraction was carried out by strictly adhering to introduced definitions and keywords during the selection and assignment of relevant results to ethical principles.

Since our aim was to focus specifically on empirical research, a presentation of the thorough analysis of theoretical literature may have fallen short. Furthermore, the broad scope of research including implant wearers’ experiences with three very different types of implants led to limitations in presenting in depth and highly differentiated comparisons and analysis within the single implant field.

## Conclusion

Ethical aspects play an important role both in implant development and care as well as in individuals’ everyday life and experiences. However, there is too little empirical research on central ethical values (autonomy, freedom, identity, participation, safety, privacy, justice, and sustainability) for wearers of CI, GI and CVI. Furthermore, there is a great imbalance in the consideration of ethical aspects in the implant-wearer’s lifeworld, not only between different kinds of implants, but also regarding the extent to which different ethical values are addressed. Besides patient autonomy and justice in terms of equitable access and non-discrimination, ethical values such as social participation and the implant-wearer’s identity, e.g., regarding the fulfilment of social norms of having a hearing, seeing or functioning body played a major role. There was little to no empirical information on privacy, safety and sustainability issues from an implant-wearer’s standpoint. Integrating patients’ perspectives and lived experiences can inform individual healthcare, healthcare programs and future research. Patient-centered approaches could benefit from an explicit and transparent ethical framework. Eventually, this requires creating awareness for cultural and identity-related issues, on the side of patients as well as healthcare professionals and implant engineers. Additionally, (technology-related) individual and organizational HL is needed to empower patients and strengthen their autonomy. Sustainability in implant care needs to be given more consideration since the implant affects an individual’s whole lifespan.

### Supplementary Information

**Additional file 1:***“Implant-related ethical aspect_scoping review_Schulz *et al*._supplementary material.doc”* includes **Supplementary material A.** Search strategy, **Supplementary material B.** List of extracted variables, **Supplementary table 1.** Number of articles per country and continent overall as well as in each implant area (data relating to Fig. 2), **Supplementary table 2.** Thematic sorting (Overview of addressed topics in selected body of literature, thematically organized alongside the fundamental ethical values), **Supplementary table 3.** Narrative Synthesis Findings (Overview of main results of the narrative synthesis of this review).

## Data Availability

The datasets used and/or analyzed during the current study are available from the corresponding author on reasonable request.

## Abbreviations

CI: Cochlear implant
MIGS: Minimally invasive glaucoma surgery
GI: Glaucoma implants
CVI: Cardiovascular implants
TAVI: Transcatheter aortic valve implantation
DM: Decision-making
SDM: Shared decision-making
QoL: Quality of life
SAVR: Surgical aortic valve replacement
HL: Health literacy
BMI: Brain-machine-interface

## Footnotes

1 Collaborative Project “RESPONSE—Partnership for Innovation in Implant Technology” funded by the ministry of education and research, Germany: https://www.innovation-strukturwandel.de/strukturwandel/de/unternehmen-region/die-initiativen/_documents/artikel/p-y/response.html

## References

[1] Vincenti V, Bacciu A, Guida M, Marra F, Bertoldi B, Bacciu S, Pasanisi E (2014). Pediatric cochlear implantation: an update. Ital J Pediatr.

[2] Colletti L, Mandalà M, Colletti V (2012). Cochlear implants in children younger than 6 months. Otolaryngol Head Neck Surg.

[3] Ansari E (2017). An Update on Implants for Minimally Invasive Glaucoma Surgery (MIGS). Ophthalmol Ther.

[4] Chakos A, Wilson-Smith A, Arora S, Nguyen TC, Dhoble A, Tarantini G (2017). Long term outcomes of transcatheter aortic valve implantation (TAVI): a systematic review of 5-year survival and beyond. Ann Cardiothorac Surg.

[5] Universität Rostock (2023). RESPONSE - Partnerschaft für Innovation in der Implantattechnologie.

[6] Woopen C, Mertz M (2014). Ethik in der Technikfolgenabschätzung: Vier unverzichtbare Funktionen. Aus Politik und Zeitgeschichte.

[7] Sparrow R (2005). Defending deaf culture: the case of cochlear implants. J pol philos.

[8] Balkany T, Hodges AV, Goodman KW (1996). Ethics of cochlear implantation in young children. Otolaryngol Head Neck Surg.

[9] Ouellette A (2010). Hearing the deaf: cochlear implants, the deaf community, and bioethical analysis. Val UL Rev.

[10] Ellis JB, Reis A-E (2008). Cochlear implantation and deaf culture: modern miracle or cultural genocide. Int J Technol Knowl Soc.

[11] Jünger S, Harzheim L, Lorke M, Woopen C. Ethische Aspekte in der Forschung und Entwicklung von sowie der Versorgung mit Implantaten. [Ethical aspects in implant research and development as well as implant care]. In: Löschner U, Siegosch F, Fleßa S, editors. Strategien der Implantatentwicklung mit hohem Innovationspotenzial: Von der Idee zur erfolgreichen Standardlösung. Wiesbaden, Germany, Heidelberg: Springer Gabler; 2021. p. 171–200. 10.1007/978-3-658-33474-1_9.

[12] Bednar K (2020). Exploring Human Nature in a Technology-Driven Society. In: IFIP International Conference on Human Choice and Computers.

[13] van Dijk W, Faber MJ, Tanke MAC, Jeurissen PPT, Westert GP (2016). Medicalisation and Overdiagnosis: what society does to medicine. Int J Health Policy Manag.

[14] Pellegrino ED (1993). The Virtues in Medical Practice.

[15] Dolezal L. Morphological freedom and medicine: constructing the posthuman body. In: Atkinson S, Macnaughton J, Richards J, editors. The Edinburgh Companion to the Critical Medical Humanities. Edinburgh: Edinburgh University Press; 2016.

[16] Brey PAE, Bottis M, Panagopoulou-Koutnatzi F (2016). Self-identity and the evaluation of medical technology. Bioethical concerns: the human face.

[17] Adler JM (2018). Bringing the (disabled) body to personality psychology: a case study of Samantha. J Pers.

[18] Hübner C, Lorke M, Buchholz A, Frech S, Harzheim L, Schulz S (2022). Health literacy in the context of implant care-perspectives of (Prospective) implant wearers on individual and organisational factors. Int J Environ Res Public Health.

[19] Mo B, Lindbæk M, Harris S (2005). Cochlear implants and quality of life: a prospective study. Ear Hear.

[20] Yamin AE (2009). Shades of dignity: exploring the demands of equality in applying human rights frameworks to health. Health Hum Rts.

[21] European Union (2012). Council of the European Union. Charter of Fundamental Rights of the European Union (2012/ C 326).

[22] World Health Organization (WHO) (1946). Constitution of the World Health Organization. Am J Public Health Nations Health.

[23] Deutscher Ethikrat (2016). Patientwohl als ethischer Maßstab für das Krankenhaus: Stellungnahme.

[24] Datenethikkommission der Bundesregierung (DEK). Gutachten der Datenethikkommission der Bundesregierung. Berlin: DEK; 2019. https://www.bmi.bund.de/SharedDocs/downloads/DE/publikationen/themen/it-digitalpolitik/gutachten-datenethikkommission.html. Accessed 9 Sep 2022.

[25] Donnelly M (2011). Healthcare Decision-Making and the Law.

[26] Christman J (2003). Autonomy in Moral and Political Philosophy.

[27] Carter I (2003). Positive and Negative Liberty.

[28] Hansson SO (2005). Implant ethics. J Med Ethics.

[29] Schwartz SJ, Zamboanga BL, Weisskirch RS (2008). Broadening the Study of the Self: Integrating the Study of Personal Identity and Cultural Identity. Soc Pers Psychol Compass.

[30] Witt K (2017). Identity change and informed consent. J Med Ethics.

[31] Woopen C, Joerden JC, Hilgendorf E, Petrillo N, Thiele F (2012). Personale Identität und Neuromodulation. Zu möglichen Auswirkungen Tiefer Hirnstimulation auf Personalität und Persönlichkeit. Menschenwürde in der Medizin: Quo vadis?.

[32] Shoemaker D (2005). Personal Identity and Ethics.

[33] Davis JL. Embodiment. In: Cockerham WC, editor. The Wiley-Blackwell encyclopedia of health, illness, behavior, and society. Chichester: Wiley-Blackwell; 2014. p. 464–468. 10.1002/9781118410868.wbehibs430.

[34] van den Hoven J, Blaauw M, Pieters W, Warnier M (2014). Privacy and Information Technology.

[35] Mohan D (2003). Introduction: safety as a human right. Health & Hum Rts.

[36] Levasseur M, Richard L, Gauvin L, Raymond E (2010). Inventory and analysis of definitions of social participation found in the aging literature: proposed taxonomy of social activities. Soc Sci Med.

[37] Campbell CS, Clark LA, Loy D, Keenan JF, Matthews K, Winograd T, Zoloth L (2007). The Bodily Incorporation of Mechanical Devices: Ethical and Religious Issues (Part 1). Camb Q Healthc Ethics.

[38] Napier AD, Ancarno C, Butler B, Calabrese J, Chater A, Chatterjee H (2014). Culture and health. Lancet.

[39] Feinsod FM, Wagner C (2008). The ethical principle of justice: The purveyor of equality. Annals long-term care.

[40] Commission on Social Determinats of Health (CSDH). Closing the gap in a generation: Health equity through action on the social determinants of health. Final report of the Commission on Social Determinants of Health. Geneva: World Health Organization (WHO); 2008.

[41] Stangl AL, Earnshaw VA, Logie CH, van Brakel WC , Simbayi L, Barré I, Dovidio JF (2019). The Health Stigma and Discrimination Framework: a global, crosscutting framework to inform research, intervention development, and policy on health-related stigmas. BMC Med.

[42] WHO Regional Office for Europe. Environmentally sustainable health systems: a strategic document. Copenhagen: World Health Organization (WHO); 2017. https://www.who.int/publications/i/item/WHO-EURO-2017-2241-41996-57723. Accessed 9 Sep 2022.

[43] Komesaroff L, Komesaroff PA, Hyde M, Clausen J, Levy N (2015). Ethical Issues in Cochlear Implantation. Handbook of neuroethics: With 31 figures and 11 tables.

[44] Colquhoun HL, Levac D, O’Brien KK, Straus S, Tricco AC, Perrier L (2014). Scoping reviews: time for clarity in definition, methods, and reporting. J Clin Epidemiol.

[45] Arksey H, O’Malley L (2005). Scoping studies: towards a methodological framework. Int j soc res methodol.

[46] Levac D, Colquhoun H, O’Brien KK (2010). Scoping studies: advancing the methodology. Implement Sci.

[47] Armstrong R, Hall BJ, Doyle J, Waters E (2011). Cochrane Update. ‘Scoping the scope’ of a cochrane review. J Public Health (Oxf).

[48] Tricco AC, Lillie E, Zarin W, O’Brien KK, Colquhoun H, Levac D (2018). PRISMA extension for scoping reviews (PRISMA-ScR): Checklist and Explanation. Ann Intern Med.

[49] Watson V, Verschuur C, Lathlean J (2016). Exploring the experiences of teenagers with cochlear implants. Cochlear Implants Int.

[50] Sach TH, Whynes DK (2005). Paediatric cochlear implantation: the views of parents. Int J Audiol.

[51] Athalye S, Mulla I, Archbold S (2014). The experiences of adults assessed for cochlear implantation who did not proceed. Cochlear Implants Int.

[52] Hallberg LRM, Ringdahl A (2004). Living with cochlear implants: experiences of 17 adult patients in Sweden. Int J Audiol.

[53] Hardonk S, Daniels S, Desnerck G, Loots G, van Hove G, van Kerschaver E (2011). Deaf parents and pediatric cochlear implantation: an exploration of the decision-making process. Am Ann Deaf.

[54] Mäki-Torkko EM, Vestergren S, Harder H, Lyxell B (2015). From isolation and dependence to autonomy - expectations before and experiences after cochlear implantation in adult cochlear implant users and their significant others. Disabil Rehabil.

[55] Dillon B, Pryce H (2020). What makes someone choose cochlear implantation? An exploration of factors that inform patient decision making. Int J Audiol.

[56] Jeffs E, Redfern K, Stanfield C, Starczewski H, Stone S, Twomey T, Fortnum H (2015). A pilot study to explore the experiences of congenitally or early profoundly deafened candidates who receive cochlear implants as adults. Cochlear Implants Int.

[57] Incesulu A, Vural M, Erkam U (2003). Children with cochlear implants: parental perspective. Otol Neurotol.

[58] Fitzpatrick EM, Jacques J, Neuss D (2011). Parental perspectives on decision-making and outcomes in pediatric bilateral cochlear implantation. Int J Audiol.

[59] Aloqaili Y, Arafat AS, Almarzoug A, Alalula LS, Hakami A, Almalki M, Alhuwaimel L (2019). Knowledge about cochlear implantation: a parental perspective. Cochlear Implants Int.

[60] Beattie RG, Ritter-Brinton K, Snart F (2000). A mother and son cochlear implant case study: making the decision twice. Adv Otorhinolaryngol.

[61] Okubo S, Takahashi M, Kai I (2008). How Japanese parents of deaf children arrive at decisions regarding pediatric cochlear implantation surgery: a qualitative study. Soc sci med.

[62] Ng ZY, Lamb B, Harrigan S, Archbold S, Athalye S, Allen S (2016). Perspectives of adults with cochlear implants on current CI services and daily life. Cochlear Implants Int.

[63] Vieira SDS, Dupas G, Chiari BM (2018). Cochlear implant: the family’s perspective. Cochlear Implants Int..

[64] Vieira SDS, Dupas G, Chiari BM (2018). Effects of cochlear implantation on adulthood. Codas.

[65] Nijmeijer HG, Keijsers NM, Huinck WJ, Mylanus EA (2021). The effect of cochlear implantation on autonomy, participation and work in postlingually deafened adults: a scoping review. Eur Arch Otorhinolaryngol.

[66] Chen S, Karamy B, Shipp D, Nedzelski J, Chen J, Lin V (2016). Assessment of the psychosocial impacts of cochlear implants on adult recipients and their partners. Cochlear Implants Int.

[67] Majorano M, Maes M, Morelli M, Bastianello T, Guerzoni L, Murri A, Cuda D (2018). Socio-emotional adjustment of adolescents with cochlear implants: Loneliness, emotional autonomy, self-concept, and emotional experience at the hospital. J Child Health Care.

[68] Wheeler A, Archbold S, Gregory S, Skipp A (2007). Cochlear implants: The young people’s perspective. J Deaf Stud Deaf Educ.

[69] Ibrahim MA (2014). The joy of cochlear implants. BMJ..

[70] Finlay L, Molano-Fisher P (2008). ‘Transforming’ self and world: a phenomenological study of a changing lifeworld following a cochlear implant. Med Health Care Philos.

[71] Preisler G, Tvingstedt A-L, Ahlström M (2005). Interviews with deaf children about their experiences using cochlear implants. Am Ann Deaf.

[72] Punch R, Hyde M (2011). Social participation of children and adolescents with cochlear implants: a qualitative analysis of parent, teacher, and child interviews. J Deaf Stud Deaf Educ.

[73] Dornhoffer J (2019). An Otologist’s Experience as a Cochlear Implant PatientThe Power of Neuroplasticity. JAMA Otolaryngol Head Neck Surg.

[74] Newberry E (2011). ‘I wish I had known to prepare for that’. Wife, mother, and patient: the impact on family dynamics post-implantation. Cochlear Implants Int.

[75] Kos M-I, Degive C, Boex C, Guyot J-P (2007). Professional occupation after cochlear implantation. J Laryngol Otol.

[76] Warner-Czyz AD, Loy B, Roland PS, Tobey EA (2013). A comparative study of psychosocial development in children who receive cochlear implants. Cochlear Implants Int.

[77] Chapman M, Dammeyer J (2017). The relationship between cochlear implants and deaf identity. Am Ann Deaf.

[78] Hilton K, Jones F, Harmon S, Cropper J (2013). Adolescents’ experiences of receiving and living with sequential cochlear implants: an interpretative phenomenological analysis. J Deaf Stud Deaf Educ.

[79] Kobosko J, Jedrzejczak WW, Pilka E, Pankowska A, Skarzynski H (2015). Satisfaction with cochlear implants in Postlingually deaf adults and its Nonaudiological predictors: psychological distress, coping strategies, and self-esteem. Ear Hear.

[80] Leigh IW, Maxwell-McCaw D, Bat-Chava Y, Christiansen JB (2009). Correlates of psychosocial adjustment in deaf adolescents with and without cochlear implants: a preliminary investigation. J Deaf Stud Deaf Educ.

[81] Sahli S, Belgin E (2006). Comparison of self-esteem level of adolescents with cochlear implant and normal hearing. Int J Pediatr Otorhinolaryngol.

[82] Dammeyer J, Chapman M, Marschark M (2018). Experience of hearing loss, communication, social participation, and psychological well-being among adolescents with cochlear implants. Am Ann Deaf.

[83] Anmyr L, Olsson M, Freijd A, Larsson K (2015). Sense of coherence, social networks, and mental health among children with a cochlear implant. Int J Pediatr Otorhinolaryngol.

[84] Muigg F, Weichbold VW, Kuehn H, Seebacher J, Galvan O (2021). Does cochlear implantation affect openness-to-experience in profound postlingual hearing loss?. J Deaf Stud Deaf Educ.

[85] Mance J, Edwards L (2012). Deafness-related self-perceptions and psychological well-being in deaf adolescents with cochlear implants. Cochlear Implants Int.

[86] Goldblat E, Most T (2018). Cultural identity of young deaf adults with cochlear implants in comparison to deaf without cochlear implants and hard-of-hearing young adults. J Deaf Stud Deaf Educ.

[87] Marschark M, Machmer E, Spencer LJ, Borgna G, Durkin A, Convertino C (2018). Language and psychosocial functioning among deaf learners with and without cochlear implants. J Deaf Stud Deaf Educ.

[88] Moog JS, Geers AE, Gustus CH, Brenner CA (2011). Psychosocial adjustment in adolescents who have used cochlear implants since preschool. Ear Hear.

[89] Wald RL, Knutson JF (2000). Deaf cultural identity of adolescents with and without cochlear implants. Ann Otol Rhinol Laryngol Suppl.

[90] Spencer LJ, Tomblin JB, Gantz BJ (2012). Growing up with a cochlear implant: education, vocation, and affiliation. J Deaf Stud Deaf Educ.

[91] Williams L (2019). Untreated severe-to-profound hearing loss and the cochlear implant situation: how policy and practice are disabling New Zealand society. N Z Med J.

[92] Bat-Chava Y, Martin D (2002). Sibling relationships for deaf children: The impact of child and family characteristics. Rehabil Psychol.

[93] Mauldin L (2019). Don’t look at it as a miracle cure: Contested notions of success and failure in family narratives of pediatric cochlear implantation. Soc Sci Med.

[94] Hallberg LRM, Ringdahl A, Holmes A, Carver C (2005). Psychological general well-being (quality of life) in patients with cochlear implants: importance of social environment and age. Int J Audiol.

[95] Choi JE, Hong SH, Moon I (2020). Academic performance, communication, and psychosocial development of Prelingual deaf children with cochlear implants in mainstream schools. J audiol otol.

[96] Steinberg A, Brainsky A, Bain L, Montoya L, Indenbaum M, Potsic W (2000). Parental values in the decision about cochlear implantation. Int J Pediatr Otorhinolaryngol.

[97] Singh U, Kapasi A, Patel N, Khandhar V, Neupane AK (2019). Expectations and experience of children with unilateral cochlear implantation: a parental perspective. Indian J Otolaryngol Head Neck Surg.

[98] Ontario Health (Quality) (2019). Minimally Invasive Glaucoma Surgery: A Budget Impact Analysis and Evaluation of Patients’ Experiences, Preferences, and Values.

[99] Cross V, Shah P, Glynn M, Chidrawar S (2009). ReGAE 5: Can we improve the surgical journey for African-Caribbean patients undergoing glaucoma filtration surgery?. Some preliminary findings Clin Ophthalmol.

[100] Foo RCM, Lamoureux EL, Wong RCK, Ho S-W, Chiang PPC, Rees G (2012). Acceptance, attitudes, and beliefs of Singaporean Chinese toward an ocular implant for glaucoma drug delivery. Invest Ophthalmol Vis Sci.

[101] Skaar E, Ranhoff AH, Nordrehaug JE, Forman DE, Schaufel MA (2017). Conditions for autonomous choice: a qualitative study of older adults’ experience of decision-making in TAVR. J Geriatr Cardiol.

[102] Ontario Health (Quality) (2020). Transcatheter Aortic Valve Implantation in Patients With Severe Aortic Valve Stenosis at Low Surgical Risk: A Health Technology Assessment.

[103] Schmied Wolfram, Schäfers Hans-Joachim, and Köllner Volker. Lebensqualität oder Lebenserwartung? Kriterien und Informationsquellen für die Entscheidungsfindung bei Patienten im Vorfeld von Aortenklappenoperationen/ Quality of life or life expectancy? Criteria and sources of information in the decision-making of patients undergoing aortic valve surgery. Z PsychosomMed Psychother. 2015:224–37. 10.13109/zptm.2015.61.3.224 26388054

[104] Olsson K, Naslund U, Nilsson J, Hornsten A (2018). Patients’ experiences of the transcatheter aortic valve implantation trajectory: a grounded theory study. Nurs Open.

[105] Bryssinck L, de Vlieger S, François K, Bové T (2021). Post hoc patient satisfaction with the choice of valve prosthesis for aortic valve replacement: results of a single-centre survey. Interact Cardiovasc Thorac Surg.

[106] Rauen JA, Rauen CA (2006). A patient’s bold voice: a journey through cardiac surgery. AACN Adv Crit Care.

[107] Lauck SB, Baumbusch J, Achtem L, Forman JM, Carroll SL, Cheung A (2016). Factors influencing the decision of older adults to be assessed for transcatheter aortic valve implantation: an exploratory study. Eur J Cardiovasc Nurs.

[108] Korteland NM, Bras FJ, van Hout FMA, Kluin J, Klautz RJM, Bogers AJJC, Takkenberg JJM (2015). Prosthetic aortic valve selection: current patient experience, preferences and knowledge. Open Heart.

[109] Mehrpoya A, Jalali R, Jalali A, Namdari M (2018). Patient experiences of living with coronary stent. J Vasc Nurs.

[110] Frankel NZ (2014). Surgical aortic valve replacement vs transcatheter aortic valve replacement: a consumer’s perspective regarding data education and transparency of hospitals. JAMA Intern Med.

[111] Baumbusch J, Lauck SB, Achtem L, O’Shea T, Wu S, Banner D (2018). Understanding experiences of undergoing transcatheter aortic valve implantation: one-year follow-up. Eur J Cardiovasc Nurs.

[112] Berg SK, Zwisler A-D, Pedersen BD, Haase K, Sibilitz KL (2013). Patient experiences of recovery after heart valve replacement: suffering weakness, struggling to resume normality. BMC Nurs.

[113] Oterhals K, Fridlund B, Nordrehaug JE, Haaverstad R, Norekvål TM (2013). Adapting to living with a mechanical aortic heart valve: a phenomenographic study. J Adv Nurs.

[114] Astin F, Horrocks J, McLenachan J, Blackman DJ, Stephenson J, Closs SJ (2017). The impact of transcatheter aortic valve implantation on quality of life: a mixed methods study. Heart Lung.

[115] Lytvyn L, Guyatt GH, Manja V, Siemieniuk RA, Zhang Y, Agoritsas T, Vandvik PO (2016). Patient values and preferences on transcatheter or surgical aortic valve replacement therapy for aortic stenosis: a systematic review. BMJ Open.

[116] Marsh K, Hawken N, Brookes E, Kuehn C, Liden B (2019). Patient-centered benefit-risk analysis of transcatheter aortic valve replacement. F1000Res.

[117] Stirrat GM, Gill R (2005). Autonomy in medical ethics after O’Neill. J Med Ethics.

[118] Entwistle VA, Carter SM, Cribb A, McCaffery K (2010). Supporting patient autonomy: the importance of clinician-patient relationships. J gen intern med.

[119] Shlobin NA, Rosenow JM (2022). Ethical considerations in the implantation of Neuromodulatory devices. Neuromodulation.

[120] Hutchison K, Sparrow R (2018). Ethics and the cardiac pacemaker: more than just end-of-life issues. Europace.

[121] Lantos JD (2012). Ethics for the pediatrician: the evolving ethics of cochlear implants in children. Pediatr Rev.

[122] Pass L, Graber AD (2015). Informed consent, deaf culture, and cochlear implants. J Clin Ethics.

[123] Hintermair M, Albertini JA (2005). Ethics, deafness, and new medical technologies. J Deaf Stud Deaf Educ.

[124] Dada T, Ramesh P, Sethi A, Bhartiya S (2020). Ethics of Glaucoma Widgets. J Curr Glaucoma Pract.

[125] van Velthoven EAM, van Stuijvenberg OC, Haselager DRE, Broekman M, Chen X, Roelfsema P (2022). Ethical implications of visual neuroprostheses-a systematic review. J Neural Eng.

[126] McCormick TR, Clausen J, Levy N (2015). Ethical Issues in Auditory Prostheses. Handbook of neuroethics: With 31 figures and 11 tables.

[127] Nunes R (2001). Ethical dimension of paediatric cochlear implantation. Theor med bioethics.

[128] Hladek GA (2002). Cochlear implants, the deaf culture, and ethics: a study of disability, informed surrogate consent, and ethnocide. Monash bioeth rev.

[129] Kermit P, Kristiansen K, Vehmas S, Shakespeare T (2008). Cochlear implants, linguistic rights and ‘open future’arguments. Arguing about disability.

[130] Grant RA, Halpern CH, Baltuch GH, O’Reardon JP, Caplan A (2014). Ethical considerations in deep brain stimulation for psychiatric illness. J Clin Neurosci.

[131] Yoon J, Lee M, Ahn JS, Oh D, Shin S-Y, Chang YJ, Cho J (2022). Development and validation of digital health technology literacy assessment questionnaire. J Med Syst.

[132] Decker M, Fleischer T (2008). Contacting the brain—aspects of a technology assessment of neural implants. Biotechnol J.

[133] Glannon W (2016). Ethical issues in neuroprosthetics. J Neural Eng.

[134] Echarte LE, García-Valdecasas M (2014). Identity and conflicts in the ethics of neural implants. Cuad bioét.

[135] Miyasaka M, Sasaki S, Tanaka M, Kikunaga J. Use of Brain-Machine Interfaces as Prosthetic Devices: An Ethical Analysis. In: The Ethical Challenges of Emerging Medical Technologies. London: Routledge; 2020. p. 237–246.

[136] Witt K, Kuhn J, Timmermann L, Zurowski M, Woopen C (2013). Deep Brain Stimulation and the Search for Identity. Neuroethics.

[137] Lipsman N, Glannon W (2013). Brain, mind and machine: what are the implications of deep brain stimulation for perceptions of personal identity, agency and free will?. Bioethics.

[138] Battaglia F (2021). Agency, Responsibility, Selves, and the Mechanical Mind. Philosophies.

[139] Lee J (2016). Cochlear implantation, enhancements, transhumanism and Posthumanism: some human questions. Sci Eng Ethics.

[140] Edelist T (2016). Capitalising on cultural dichotomies: Making the ‘right choice’ regarding cochlear implants. Soc Theory Health.

[141] Levy N (2002). Reconsidering cochlear implants: the lessons of Martha’s Vineyard. Bioethics.

[142] Maia TG (2020). Cochlear implants in congenitally deaf children: a discussion built on rights-based arguments. Am Ann Deaf.

[143] Besmer K. Embodying a Translation Technology. Techne: Res Philos. 2012;16:296–316. 10.5840/techne201216319.

[144] Barfield W, Williams A. Cyborgs and Enhancement Technology. Philosophies. 2017;2:4. 10.3390/philosophies2010004.

[145] Vinod K, Gedde SJ (2021). Safety profile of minimally invasive glaucoma surgery. Curr Opin Ophthalmol.

[146] Ontario Health (Quality) (2021). iStent for Adults With Glaucoma: A Health Technology Assessment. Ont Health Technol Assess Ser.

[147] Zaman A, de Winter RJ, Kogame N, Chang CC, Modolo R, Spitzer E (2019). Safety and efficacy of a sirolimus-eluting coronary stent with ultra-thin strut for treatment of atherosclerotic lesions (TALENT): a prospective multicentre randomised controlled trial. Lancet.

[148] Das S, Siroky GP, Lee S, Mehta D, Suri R (2021). Cybersecurity: The need for data and patient safety with cardiac implantable electronic devices. Heart Rhythm.

[149] Taitsman JK, Grimm CM, Agrawal S (2013). Protecting patient privacy and data security. N Engl J Med.

[150] Shen N, Bernier T, Sequeira L, Strauss J, Silver MP, Carter-Langford A, Wiljer D (2019). Understanding the patient privacy perspective on health information exchange: a systematic review. Int J Med Inform.

[151] Camara C, Peris-Lopez P, Tapiador JE (2015). Security and privacy issues in implantable medical devices: a comprehensive survey. J Biomed Inform.

[152] Capkun S, Bodmer D. On the security and privacy risks in cochlear implants. Technical Report / ETH Zurich, Department of Computer Science 2010. 10.3929/ETHZ-A-006862326.

[153] Berghold A, Hübner C, Schmitz-Luhn B, Woopen C (2022). Tech Giants Healthcare.

[154] Gaobotse G, Mbunge E, Batani J, Muchemwa B (2022). Non-invasive smart implants in healthcare: Redefining healthcare services delivery through sensors and emerging digital health technologies. Sens Int..

[155] Caplan AL, Parent B (2017). The ethical challenges of emerging medical technologies.

[156] Green J, Siddall H, Murdoch I (2002). Learning to live with glaucoma: a qualitative study of diagnosis and the impact of sight loss. Soc sci med.

[157] Jin S, Trope GE, Buys YM, Badley EM, Thavorn K, Yan P (2019). Reduced social participation among seniors with self-reported visual impairment and glaucoma. PLoS One.

[158] Yang Y, Trope GE, Buys YM, Badley EM, Gignac MAM, Shen C, Jin Y-P (2016). Glaucoma severity and participation in diverse social roles: does visual field loss matter?. J Glaucoma.

[159] Liu XL, Rosa-Lugo LI, Cosby JL, Pritchett CV (2021). Racial and insurance inequalities in access to early pediatric cochlear implantation. Otolaryngol Head Neck Surg.

[160] Bhartiya S (2020). Patient Centricity and the Ethics of Glaucoma Care. J Curr Glaucoma Pract.

[161] Hutchison K (2019). Gender bias in medical implant design and use: a type of moral aggregation problem?. Hypatia.

[162] McKinnon BJ (2013). Cochlear implant programs: balancing clinical and financial sustainability. Laryngoscope.

[163] Yadav D, Garg RK, Ahlawat A, Chhabra D (2020). 3D printable biomaterials for orthopedic implants: Solution for sustainable and circular economy. Resour Policy.

[164] Parvinkal S, Pardeep K (2018). An overview of biomedical materials and techniques for better functional performance, life, sustainability and biocompatibility of orthopedic implants. Indian J Sci Technol.

[165] Ruiwale VV, Sambhe RU (2015). Application of additive manufacturing technology for manufacturing medical implants: a review. Int J Mech Enig Technol.

[166] Salmi M, Tuomi J, Paloheimo K-S, Björkstrand R, Paloheimo M, Salo J (2012). Patient-specific reconstruction with 3D modeling and DMLS additive manufacturing. Rapid Prototyp J.

[167] Jang J, Jang JH, Choi H (2017). Biomimetic artificial basilar membranes for next-generation cochlear implants. Adv Healthcare Mater.

[168] Courtine G, Bloch J (2015). Defining ecological strategies in neuroprosthetics. Neuron.

[169] Trayanova N (2019). From genetics to smart watches: developments in precision cardiology. Nat Rev Cardiol.

[170] Lorke M, Hübner C, Schulz S, Woopen C. Lernmodul: Implantat-Ethik. 2022. https://response-elearning.de/implantat-ethik?sl=prv.

[171] van den Hoven J. The design turn in applied ethics. In: van den Hoven J, Miller S, Pogge T, editors. Designing in Ethics. Cambridge: Cambridge University Press; 2017. p. 11–31.

