# Supplementary material for: Patient-centered empirical research on ethically relevant psychosocial and cultural aspects of cochlear, glaucoma and cardiovascular implants – a scoping review
Source: BMC Med Ethics. 2023 Aug 28;24:68. doi: 10.1186/s12910-023-00945-6 (PMC10464431; doi:10.1186/s12910-023-00945-6)
Supplement: Supplementary file 1 — Additional file 1:“Implant-related ethical aspect_scoping review_Schulz et al._supplementary material.doc” includes Supplementary material A. Search strategy, Supplementary material B. List of extracted variables, Supplementary table 1. Number of articles per country and continent overall as well as in each implant area (data relating to Fig. 2), Supplementary table 2. Thematic sorting (Overview of addressed topics in selected body of literature, thematically organized alongside the fundamental ethical values), Supplementary table 3. Narrative Synthesis Findings (Overview of main results of the narrative synthesis of this review). [file 12910_2023_945_MOESM1_ESM.docx]

**Patient-centered empirical research on ethically relevant psychosocial and cultural aspects of cochlear, glaucoma and cardiovascular implants – a scoping review**

**Supplementary material A - Search Strategy** (Pubmed, date of literature search: 27.04.2022)

((((((( "Cochlear Implants/economics"[Mesh] OR "Cochlear Implants/education"[Mesh] OR "Cochlear Implants/ethics"[Mesh] OR "Cochlear Implants/psychology"[Mesh] ) OR "Aortic Valve"[Mesh]) OR ("Heart Valve Prosthesis Implantation/economics"[Mesh] OR "Heart Valve Prosthesis Implantation/education"[Mesh] OR "Heart Valve Prosthesis Implantation/ethics"[Mesh] OR "Heart Valve Prosthesis Implantation/psychology"[Mesh] )) OR ("Defibrillators/ethics"[Mesh] OR "Defibrillators/psychology"[Mesh] )) OR ( "Pacemaker, Artificial/ethics"[Mesh] OR "Pacemaker, Artificial/psychology"[Mesh] )) OR ( "Glaucoma Drainage Implants/economics"[Mesh] OR "Glaucoma Drainage Implants/psychology"[Mesh] )) OR ("aortic valve implantation" OR "artificial heart valve" OR "aortic valve replacement" OR "coronary stent" OR (glaucoma AND surgery)) AND (ethic* OR “quality of life” OR attitude OR perception OR perspective OR experience OR cultur* OR ethnicity OR respons*)) AND (economics OR psycho* OR decision-making OR “health literacy” OR qualitative OR “health services” OR compliance OR adherence OR social OR personal identity OR education OR lived experience OR health technology assessment OR dignity OR autonomy OR freedom OR liberty OR self-determination OR privacy OR insecurity OR security OR safety OR justice OR inequality OR equality OR democracy OR participation OR health OR integrity OR value OR norms OR solidarity OR discrimination OR sustainability OR stigmatisation) NOT (dental OR medical OR breast OR microchip OR neural OR disease)

Limitations: German, English, published since 2000

**Supplementary material B - List of extracted variables.**

- study authors
- year of publication
- journal
- location of publication
- implant field
- research aim
- methodological approach
- data collection method and outcomes (according to applied specific questionnaires, surveys, single items, (sub)themes, (sub)categories, (sub)headings in results section or interview topic guide, eligibility criteria (in the case of reviews))
- main results

**Supplementary table 1 - Number of articles per country and continent overall as well as in each implant area (data relating to Figure 2)**

| Supplementary table 1. Number of articles per country and continent overall as well as in each implant area | | | | | |
| --- | --- | --- | --- | --- | --- |
| **Continent** | **Country** | **N** | **Cochlear Implants** | **Cardiovascular Implants** | **Glaucoma Implants** |
| **Asia** | Turkey (Ankara) | 2 | Incesulu et al. 2003; Sahli & Belgin 2006; (n=2) |  |  |
|  | India | 1 | Singh et al. 2019; (n=1) |  |  |
|  | Iran | 1 |  | Mehrpoya et al. 2018; (n=1) |  |
|  | Israel | 1 | Goldblat & Most 2018; (n=1) |  |  |
|  | Japan | 1 | Okubo et al. 2008; (n=1) |  |  |
|  | Saudi Arabia | 1 | Aloqaili et al. 2019; (n=1) |  |  |
|  | Singapore | 1 |  |  | Foo et al. 2012; (n=1) |
|  | South Korea | 1 | Choi et al. 2020; (n=1) |  |  |
| **Australia** | Australia | 2 | Beattie et al. 2000; Punch & Hyde 2011; (n=2) |  |  |
|  | New Zealand | 1 | Williams 2019; (n=1) |  |  |
| **Europe** | UK | 14 | Athalye et al. 2014; Dillon & Pryce 2020; Finlay & Molano-Fisher 2008; Hilton et al. 2013; Jeffs et al. 2015; Kos et al. 2007; Mance & Edwards 2012; Newberry 2011; Ng et al. 2016; Sach & Whynes 2005; Watson et al. 2016; Wheeler et al. 2007; (n=12) | Astin et al. 2017; (n=1) | Cross et al. 2009; (n=1) |
|  | Sweden | 6 | Anmyr et al. 2015; Hallberg et al. 2005; Maki-Torkko et al. 2015; Preisler et al. 2005; Hallberg & Rindahl 2004; (n=5) | Olsson et al. 2018; (n=1) |  |
|  | Denmark | 3 | Chapman & Dammeyer 2017; Dammeyer et al. 2018; (n=2) | Berg et al. 2013; (n=1) |  |
|  | Norway | 2 |  | Oterhals et al. 2013; Skaar et al. 2017; (n=2) |  |
|  | Belgium | 2 | Hardonk et al. 2011; (n=1) | Bryssinck et al. 2021; (n=1) |  |
|  | Netherlands | 2 | Nijmeijer et al. 2021; (n=1) | Korteland et al. 2015; (n=1) |  |
|  | Austria | 1 | Muigg et al. 2021; (n=1) |  |  |
|  | Germany | 1 |  | Schmied et al. 2015; (n=1) |  |
|  | Italy | 1 | Majorano et al. 2018; (n=1) |  |  |
|  | Poland | 1 | Kobosko et al. 2015; (n=1) |  |  |
| **North America** | USA | 15 | Adler 2018; Bat-Chava & Martin 2002; Dornhoffer 2019; Ibrahim 2014; Leigh et al. 2009; Marschark et al. 2018; Mauldin 2019; Moog et al. 2011; Spencer et al. 2012; Steinerg et al. 2020; Wald & Knutson 2000; Warner-Czyz et al. 2013; (n=12) | Frankel 2014; Marsh et al. 2019; Rauen & Rauen 2006; (n=3) |  |
|  | Canada | 8 | Chen et al. 2016; Fitzpatrick et al. 2011; (n=2) | Baumbusch et al. 2018; Lauck et al. 2016; Lytvyn et al. 2016; Ontario Health 2020; (n=4) | CADTH 2019; (n=1) |
| **South America** | Brazil | 2 | Vieira et al. 2018a; Vieira et al. 2018b; (n=2) |  |  |

**Supplementary table 2 - Thematic sorting**

| **Supplementary table 2 - Overview of addressed topics in selected body of literature** | | | |
| --- | --- | --- | --- |
| **Fundamental ethical value(s)** | **Psychosocial and cultural aspects (classified alongside fundamental ethical values by authors of this review)** | | |
|  | **CI (n=50)** | **GI (n=3)** | **CVI (n=16)** |
| *Autonomy* | **Self-determined and informed decision-making**  QUALITATIVE Information access (Athalye et al., 2014; Ng et al., 2016), information needs & sources (Dillon & Pryce, 2020), consideration of risk (Dillon & Pryce, 2020), information needs (Fitzpatrick et al., 2011), decision-making process (Fitzpatrick et al., 2011), information (Mäki-Torkko et al., 2015), information and knowledge about hearing loss and assistive technology (Hardonk et al., 2011), understanding of technology (Wheeler et al., 2007), making the choice (Sach & Whynes, 2005), making the decision to implant (Wheeler et al., 2007), mothers’ decision for her son, mother’s personal decision (Beattie et al., 2000); *mentioned*: evaluation of provided information & sources (Sach & Whynes, 2005), pre-operative information (Watson et al., 2016), personal motivation (Jeffs et al., 2015), had to make a decision (Ibrahim, 2014)  QUANTITATIVE Sources of information (Aloqaili et al., 2019), quality of information (Aloqaili et al., 2019), sufficiency of information (Aloqaili et al., 2019), information gathering process and experience (Aloqaili et al., 2019), the implant center provided sufficient information about the surgery/ implant use (Incesulu et al., 2003), decision to implant (Incesulu et al., 2003)  **Shared decision making and patient-doctor-relationship**  QUALITATIVE Professional advice and referral for CI and hearing aids (Hardonk et al., 2011), blind faith and trust (Beattie et al., 2000); *mentioned*: given no choice, simply acquiescing with referral decision of child’s consultant (Sach & Whynes, 2005)  QUANTITATIVE Impact of information source (health professionals) on parental decision (Aloqaili et al., 2019)  **Autonomy and (in)dependence**  QUALITATIVE Increase of independence and confidence (Ng et al., 2016), fear-autonomy (Mäki-Torkko et al., 2015), less dependency on others (Vieira et al., 2018a); *mentioned:* improved independence/less dependency (Hallberg & Ringdahl, 2004), increases in independence (Sach & Whynes, 2005), more independence and autonomy (Vieira et al., 2018a), being able to hear provides gains in autonomy and independence (Vieira et al., 2018b), CI enabled to feel more independent (Watson et al., 2016), desire to be able to live independently (Dillon & Pryce, 2020), parental overprotection and dependence (Vieira et al., 2018a), understanding information & knowledge (Vieira et al., 2018b), feeling more informed (Hallberg & Ringdahl, 2004)  QUANTITATIVE Emotional autonomy (Majorano et al., 2018), feeling more assertive (Chen et al., 2016)  REVIEW Autonomy (Nijmeijer et al., 2021)  **Self-determined device usage**  QUALITATIVE *mentioned*: implant was so useful that participant felt dependent on it (Wheeler et al., 2007), being totally reliant on implant (Incesulu et al., 2003), lack of technological understanding (Wheeler et al., 2007), opportunity to switch off hearing (Finlay & Molano-Fisher, 2008; Preisler et al., 2005), anxiety regarding device failure (Hallberg & Ringdahl, 2004; Incesulu et al., 2003; Watson et al., 2016; Wheeler et al., 2007), adapting clothes and accessories to facilitate use of implant system, stocking spare parts to prevent deprivation in case of device break (Vieira et al., 2018a), progressive telephone use starting with closest people to gain confidence and tranquility (Vieira et al., 2018b), need to adapt behavior to new sounds (Watson et al., 2016), challenge of learning how to pick up and distinguish sounds, to use ears first (Finlay & Molano-Fisher, 2008), long-lasting audiovisual training (Hallberg & Ringdahl, 2004), switching hearing device to proper position using remote control (Ibrahim, 2014), repeated adjustments to programming (Dornhoffer, 2019)  **Surrogate decision-making**  QUALITATIVE Surrogate DM (Hardonk et al., 2011), Reluctance and the necessity for surrogate DM (Okubo et al., 2008)  QUANTITATIVE I am concerned that my child could blame me for this decision in the future (Incesulu et al., 2003) | **Self-determined and informed decision-making**  QUALITATIVE Surgical decision-making (Ontario Health (Quality), 2019 Dec), *mentioned:* participatory decision-making (Cross et al., 2009)  QUANTIATIV Beliefs about medicine (Foo et al., 2012), illness perception (Foo et al., 2012), medication adherence (Foo et al., 2012) (as factors of decision-making)  **Shared decision-making and patient-doctor-relationship**  QUALITATIVE Meeting the glaucoma surgeon (Cross et al., 2009); *mentioned:* trust between physician and patient (Ontario Health (Quality), 2019 Dec)  **Limited options**  QUALITATIVE *mentioned*: perceived necessity of procedure to prevent the feared blindness (Ontario Health (Quality), 2019 Dec)  **Autonomy and (in)dependence** QUALITATIVE *mentioned:* eye sight essential to maintain individuality and not be dependent on others (Ontario Health (Quality), 2019 Dec) | **Self-determined and informed decision-making**  QUALITATIVE Information needs (Baumbusch et al., 2018), contact with healthcare staff (Berg et al., 2013), influence on providers on expectations, unexpected reality (Baumbusch et al., 2018), sources of expertise (Lauck et al., 2016), lack of knowledge (Mehrpoya et al., 2018), the unaware patient (Oterhals et al., 2013), deliberately taking the chance (Skaar et al., 2017); *mentioned:* knowledge about anticoagulant therapy (Rauen & Rauen, 2006), provided risk information (Skaar et al., 2017), consumer’s perspective regarding data education and transparency of hospitals (Frankel, 2014), competent patients were well informed about their disease and its implications (Oterhals et al., 2013)  QUANTIATIVE Patient knowledge about prosthesis and patient numeracy (Korteland et al., 2015), sources of information (Schmied Wolfram, Schäfers Hans-Joachim, and Köllner Volker, 2015)  REVIEW 1 Values and preferences regarding decision for treatment (Lytvyn et al., 2016)  **Shared decision-making and patient-doctor-relationship** QUALITATIVE Formal support: physician navigator (Lauck et al., 2016), autonomous trust in their doctors (Skaar et al., 2017), being respected and participating (Olsson et al., 2018), having confidence (Olsson et al., 2018); *mentioned:* physician did not always have sufficient knowledge of condition (Oterhals et al., 2013), trust in physician establishes confidence in decision-making (Ontario Health (Quality), 2020 Nov)  QUANTITATIVE Patient-doctor-relationship in the choice of prosthesis (Bryssinck et al., 2021), patient preferences on participation in decision-making (Korteland et al., 2015), shared decision-making (Schmied Wolfram, Schäfers Hans-Joachim, and Köllner Volker, 2015)    **Limited options**  QUALITATIVE Limited options (Baumbusch et al., 2018), *mentioned*: something had to be done (Skaar et al., 2017), having little choice (Astin et al., 2017)  **Personal value of self-determination**  QUALITATIVE Fundamental self-determination based on personal identity (Skaar et al., 2017); *mentioned*: wanting to be relieved of the decision (Rauen & Rauen, 2006) |
| *Freedom* | **Limitations related to hearing loss**  QUANTITATIVE Feelings of limitation on account of hearing loss (Chapman & Dammeyer, 2017)  **(Re-)Gained abilities in daily life**  QUALITATIVE Enjoying speaking on the telephone, listening to music, and other entertainment (Vieira et al., 2018b), to hear (Preisler et al., 2005), music appreciation (Dornhoffer, 2019); *mentioned*: using normal phone (Newberry, 2011), ability to now enjoy ostensibly mundane activities (Sach & Whynes, 2005), music sounds like music again (Ibrahim, 2014), music (Jeffs et al., 2015), being able to hear provides gains in freedom, practicality and agility (Vieira et al., 2018b), playing an instrument (Vieira et al., 2018a), television (Dornhoffer, 2019), having more fun listening to music, playing, watching television (Incesulu et al., 2003)  QUANTIATIVE Watching TV more (Chen et al., 2016), enjoy music more (Chen et al., 2016), fewer problems with leisure activities (Chen et al., 2016), fewer problems functioning alone at home (Chen et al., 2016), hearing no longer limit personal/social life (Chen et al., 2016), non-professional activities (Kos et al., 2007), participating in activities (Warner-Czyz et al., 2013), using a telephone successfully (Warner-Czyz et al., 2013)  **Implant-related limitations in daily life**  QUALITATIVE Restrictions on daily activities (Okubo et al., 2008); *mentioned:* limitations and restrictions imposed by the device (Vieira et al., 2018a), hinders her doing some things since its quite bulky to carry around’ (Sach & Whynes, 2005), frustration towards the hardware and fragility of the device (Watson et al., 2016) restrictions taking care of the device (Warner-Czyz et al., 2013), batteries as major problem (Mäki-Torkko et al., 2015), responsibility of device management (Watson et al., 2016), restricted from contact sports (Hilton et al., 2013) | **Impact of glaucoma on daily lives** QUALITATIVE Impact (daily life: reading, driving, shopping) (Ontario Health (Quality), 2019 Dec), eye drops (Ontario Health (Quality), 2019 Dec); *mentioned*: eyesight essential to maintaining activity and QoL (Ontario Health (Quality), 2019 Dec)  QUANTITATIVE Adherence problems (Foo et al., 2012) | **Limited freedom in daily life pre-implant**  QUALITATIVE Limited life (functional restrictions) (Astin et al., 2017), symptom burden (Lauck et al., 2016); *mentioned:* increased dependence on other people to get through daily activities (Ontario Health (Quality), 2020 Nov)  **(Re-)gained freedom and independence**  QUALITATIVE Extended life (Astin et al., 2017), being independent (Olsson et al., 2018), achieving normality (Baumbusch et al., 2018); *mentioned:* lost independence/increased levels of dependence (Astin et al., 2017), feeling normal and in that process to regain independence was the goal (Berg et al., 2013), symptoms that constricted life before surgery disappeared (Oterhals et al., 2013), ’bridge’ to other health interventions (Astin et al., 2017), still faced complex health challenges (Baumbusch et al., 2018)  QUANTIATIVE Experience improvements in your degree of independence and ability to engage in activities of daily living (Marsh et al., 2019)  **Implant-related limitations in daily life**  QUALITATIVE Taking warfarin influences everyday life (Oterhals et al., 2013), life on warfarin (Rauen & Rauen, 2006)  QUANTITATIVE Interferences with the activities of daily life related to the prosthesis (Bryssinck et al., 2021) |
| *Identity* | **Self-relation**  QUALITATIVE Increase of independence and confidence (Ng et al., 2016), improved confidence (Hilton et al., 2013), strengthening of self-worth (Hallberg & Ringdahl, 2004); *mentioned*: increased self-confidence (Hallberg & Ringdahl, 2004), confidence grew following initial tuning (Newberry, 2011), increases in confidence (Sach & Whynes, 2005), generating confidence (Vieira et al., 2018b), CI enabled to feel more confident (Watson et al., 2016), development of confidence (Punch & Hyde, 2011), increasing self-esteem (Vieira et al., 2018b), increased perceived self-value (Mäki-Torkko et al., 2015), increase in confidence (Jeffs et al., 2015), self-concept issues (Dammeyer et al., 2018)  QUANTIATIVE Self-confidence (Incesulu et al., 2003), feel self-confidence improves (Chen et al., 2016), self-esteem (Kobosko et al., 2015; Moog et al., 2011; Sahli & Belgin, 2006), self-esteem and social relationships (Choi et al., 2020), self-perception (Leigh et al., 2009; Mance & Edwards, 2012), self-concept (Majorano et al., 2018), sense of coherence (Anmyr et al., 2015)  **Personality**  QUALITATIVE *mentioned*: CI played part in more outgoing “personality” (Punch & Hyde, 2011)  QUANTIATIVE Personality factors (Neo-FFI) (Muigg et al., 2021)  **Appearance of CI & embodiment**  QUALITATIVE Looking different (Hilton et al., 2013); *mentioned*: self-conscious about appearance of implant equipment (Punch & Hyde, 2011) hiding the device (Watson et al., 2016), newer models not as noticeable (Preisler et al., 2005), natural part of life (Preisler et al., 2005), embarrassing prosthetics (Sach & Whynes, 2005), body/perception unity (Finlay & Molano-Fisher, 2008),  QUANTIATIVE embarrassment about CI looks (Warner-Czyz et al., 2013), physical self-concept (Majorano et al., 2018),  **Life-changing experience**  QUALITATIVE Coming back to life (Hallberg & Ringdahl, 2004), significant revelation (Hallberg & Ringdahl, 2004), having one’s life restore (Vieira et al., 2018a), living under new perspective (Vieira et al., 2018b), the meaning of hearing in life (Vieira et al., 2018b); *mentioned*: being a whole new human being (Finlay & Molano-Fisher, 2008)  **Awareness of disability**  QUALITATIVE Normal vs disability (Hilton et al., 2013); *mentioned:* created an awareness of disability (Sach & Whynes, 2005), better realizing ability (Finlay & Molano-Fisher, 2008), how other people simply can hear without thinking about it (Adler, 2018)  QUANTITATIVE Perceive as hearing-impaired now (Chen et al., 2016)  **(Deaf) Identity**  QUALITATIVE Bicultural identity (Hilton et al., 2013), belonging to both worlds (Watson et al., 2016), disability identity (Adler, 2018), identity (Jeffs et al., 2015; Preisler et al., 2005; Wheeler et al., 2007), social identity (Dillon & Pryce, 2020), being a hearing person (Hilton et al., 2013)  QUANTIATIVE Deaf identity (Chapman & Dammeyer, 2017; Goldblat & Most, 2018; Spencer et al., 2012; Wald & Knutson, 2000), deaf acculturation (Leigh et al., 2009; Marschark et al., 2018), group identification (Moog et al., 2011), deafness-related self-perceptions (Mance & Edwards, 2012) | **xx** | **Self-relation**  QUALITATIVE *mentioned:* feeling worthless and burdensome (Astin et al., 2017)  **Physical appearance/embodiment**  QUALITATIVE Hides surgery-related scars (Oterhals et al., 2013), disturbed body (Berg et al., 2013); *mentioned*: sense of physical impairment (Mehrpoya et al., 2018)  **Life-changing experience**  QUALITATIVE Changed life (Astin et al., 2017), returning to life (Olsson et al., 2018)  **Personality**  QUALITATIVE *mentioned*: change of personality (Mehrpoya et al., 2018) |
| *Safety & security* | **Personal safety**  QUALITATIVE *mentioned*: hear environmental sounds that could function as warning signals (Preisler et al., 2005), safety against environmental hazards (Vieira et al., 2018b), improved hearing leads to improved personal safety (Sach & Whynes, 2005), untreated condition means not being able to keep oneself safe from bicycles, trains and motor cars (Williams, 2019), increased independence relevant regarding safety (Ng et al., 2016)  QUANTIATIVE I can let my child play outside since he can hear the traffic and other sounds (Incesulu et al., 2003), hear cars approach in traffic (Chen et al., 2016)  **Feeling safe**  QUALITATIVE *mentioned:* concerns about having such an implant inside one’s head (Wheeler et al., 2007) | **Personal safety**  QUALITATIVE *mentioned:* having vision enables personal safety (Ontario Health (Quality), 2019 Dec), fear of losing eyesight (Ontario Health (Quality), 2019 Dec) | **Feeling safe**  QUALITATIVE Facing mortality (Astin et al., 2017), feeling threatened – facing death (Olsson et al., 2018), feeling safe and secure (Astin et al., 2017), the worried patient (Oterhals et al., 2013); *mentioned*: information and training instructions made participants feel safe (Oterhals et al., 2013), heart disease rehabilitation program contributes to sense of safety (Berg et al., 2013), thinking about closeness of death (Berg et al., 2013), anxiety regarding implant functioning and stent lifetime (Mehrpoya et al., 2018) |
| *Privacy* | **Privacy**  QUALITATIVE *mentioned:* CI gives back privacy (Vieira et al., 2018b), wishing implant to be less noticeable (Watson et al., 2016), hiding CI (Dammeyer et al., 2018; Hilton et al., 2013) | xx | **Privacy**  QUALITATIVE *mentioned*: strategies to drown out sound of valve and hoping to shift people’s attention (Oterhals et al., 2013) |
| *Participation* | **Interpersonal relationships**  QUALITATIVE Family and social life (Ng et al., 2016), sibling relationships (Bat-Chava & Martin, 2002), sociality (Finlay & Molano-Fisher, 2008), living social life (Mäki-Torkko et al., 2015), peer interaction (Preisler et al., 2005), friendship patterns, awareness of nuances in social interactions (Punch & Hyde, 2011), impact on family life (Sach & Whynes, 2005), more efficient communication and social interaction (Vieira et al., 2018b); *mentioned:* impact on family dynamics and colleagues (Newberry, 2011), being able to follow conversation and participate in them (Ibrahim, 2014), nature of communication (Mauldin, 2019)  QUANTIATIVE Social relationships (Incesulu et al., 2003), friendships (Dammeyer et al., 2018; Incesulu et al., 2003), self-esteem and social relationships (Choi et al., 2020), socializing with deaf and hearing peers (Leigh et al., 2009), social networks (Anmyr et al., 2015), feeling less left out, more at ease interacting with other people, make contact with other people more easily, less anxious talking to strangers (Chen et al., 2016), feeling more sociable, interact more easily (Watson et al., 2016), friendships (Moog et al., 2011)  **Education, employment**  QUALITATIVE Education (Incesulu et al., 2003; Sach & Whynes, 2005; Wheeler et al., 2007), positive effects on education and employment (Ng et al., 2016), expectations regarding education and employment (Hardonk et al., 2011); *mentioned*: more involved and able to perform work tasks just like anyone else (Mäki-Torkko et al., 2015), educational benefits of better hearing (Hilton et al., 2013)  QUANTIATIVE Professional activities (Kos et al., 2007), challenges of working with hearing peers (Chapman & Dammeyer, 2017), academic performance (Choi et al., 2020), education (Hallberg et al., 2005), fewer problems with work or studies (Chen et al., 2016)  REVIEW Work (Nijmeijer et al., 2021)  **Social participation and acceptance in a hearing world**  QUALITATIVE Increasing social participation (Hallberg & Ringdahl, 2004), communication mode and social participation (Hardonk et al., 2011), social deafness (Punch & Hyde, 2011), alienation-normality (Mäki-Torkko et al., 2015), feelings of isolation and difference in a hearing world (Hilton et al., 2013), inclusion and integration, living context and support (Dillon & Pryce, 2020); *mentioned:* greater connected ness to hearing world (Jeffs et al., 2015), fear of environment being bothered by hearing difficulties (Hallberg et al., 2005), concerns of looking different (Finlay & Molano-Fisher, 2008), confidence and supportive friends facilitate social participation (Punch & Hyde, 2011), being able to function more like a hearing person (Watson et al., 2016)  QUANTITATIVE Social participation and friendships (Dammeyer et al., 2018), social skills and participation (Singh et al., 2019), friendships and social participation (Chapman & Dammeyer, 2017), peer acceptance (Wald & Knutson, 2000), acceptance by peers (Warner-Czyz et al., 2013), perceived acceptance by deaf and hearing peers (Leigh et al., 2009), perceived availability of social support (Hallberg et al., 2005), similarity to peers and well-being (Mance & Edwards, 2012) preferences regarding social life (Steinberg et al., 2000)  REVIEW Participation (Nijmeijer et al., 2021) | **Social participation**  QUALITATIVE *mentioned*: ability to live a life with meaning was dependent upon retaining vision, pre-existing perceptions what it means to be blind/fearing blindness connected to fears of being unable to engage in the world (Ontario Health (Quality), 2019 Dec)  **Overlooked condition**  QUALITATIVE *mentioned:* invisible condition, overlooked and ignored by others (Ontario Health (Quality), 2019 Dec) | **Social participation**  QUALITATIVE Limited life (social restrictions) (Astin et al., 2017), being isolated and dependent (Olsson et al., 2018), obligation and responsibilities (Lauck et al., 2016); *mentioned:* choosing intervention as obligation to relatives (Skaar et al., 2017)  **Social acceptance and support**  QUALITATIVE Anxiety of being accepted (Mehrpoya et al., 2018), telling friends and family (Rauen & Rauen, 2006) |
| *Justice* | **Equal access**  QUALITATIVE Living conditions and support (Dillon & Pryce, 2020); *mentioned:* having to fight for services (Sach & Whynes, 2005)  **Cost**  QUALITATIVE Cost of CI upgrades (Okubo et al., 2008); *mentioned*: expense of rehabilitation (Vieira et al., 2018a), cost of batteries (Mäki-Torkko et al., 2015), out-of-pocket contributions to implantation and device cost (Ibrahim, 2014), cost of traveling to implant center (Incesulu et al., 2003)  **Institutional discrimination**  QUALITATIVE Racism: structural “failure” and minority families (Mauldin, 2019); *mentioned:* access to CI for Maori minority (Williams, 2019), having to fight for services (Sach & Whynes, 2005)  **Discrimination & stigmatization**  QUALITATIVE Unfairness of prejudice and deafness (Hilton et al., 2013); *mentioned*: shame and stigma of disability (Vieira et al., 2018b), experiences of discrimination (Dillon & Pryce, 2020), fear of prejudice and social discrimination (Vieira et al., 2018a)  QUANTITATIVE Feeling of being discriminated against on account of hearing loss (Chapman & Dammeyer, 2017), well-being related to bullying (Dammeyer et al., 2018) | **Cost associated with glaucoma treatment**  QUALITATIVE *mentioned:* cost associated with glaucoma treatments including eye drops (Ontario Health (Quality), 2019 Dec) | **Equal access**  QUALITATIVE Address unique needs of patients living at a distance from procedure site (Baumbusch et al., 2018), logistical barriers and facilitators (Lauck et al., 2016), access (Ontario Health (Quality), 2020 Nov)  **Cost**  QUALITATIVE Economic support (Mehrpoya et al., 2018), financial barriers (Ontario Health (Quality), 2020 Nov), *mentioned:* personal cost (Lauck et al., 2016) |
| *Sustainability* | **Future planning**  QUALITATIVE Future planning (Dillon & Pryce, 2020); *mentioned:* confidence in future technical science (Hallberg & Ringdahl, 2004) | xx | xx |
| *Notes.* Topics derived from applied specific questionnaires, surveys, single items, (sub)themes, (sub)categories, (sub)headings in results section or topic guide, eligibility criteria (in the case of reviews). Otherwise, relevant topics mentioned in articles listed as “*mentioned*”. xx – no relevant data available | | | |

**Supplementaty table 3 – Narrative Synthesis Findings**

| Supplementary table 3*.* Overview of the main results of narrative synthesis of this review | | |
| --- | --- | --- |
| CI (n=50) | **GI (n=3)** | **CVI (n=16)** |
| *Autonomy* | | |
| - Perceptions of lacking or insufficient information, one-sided or biased professionals’ advice, pressure to decide for an implant, emotional burden aggravate autonomous DM - more autonomous, independent and self-determined life through CI; - Self-determined advice usage requires understanding of technology, acquiring skill and getting used to; - surrogate DM; | - Trusting PDR, severity of symptoms and fear of blindness relevant factors in DM | - DM as balancing act between dependence on physician and autonomous appraisal capacity; - Importance of participatory DM; - In case of emergency procedure more information desired after implantation; - Implantation as only option; - Importance of making decision on their own vs. wishing to be relieved of decision |
| *Freedom* | | |
| - Expanded freedom through new or refined abilities (e.g., telephone use, listening to music) lowering restrictions imposed by hearing loss; - Restrictions imposed by CI in terms of damage prevention or implant management | - Restrictions imposed by pharmacotherapy regimen - Maintaining activity and QoL through procedure | - Expanded freedom of action through reduced symptom burden after implantation - Limitations in daily life even after implantation due to persisting comorbidities; - Restrictions imposed by anticoagulation medication |
| *Identity* | | |
| - Impact of CI on self-relation in terms of increased confidence, self-worth, self-acceptance, empowerment; - Difficulties in embodiment of CI; - Existential and transformational impact of CI; - Variability in impact of CI on disability and cultural identity; | x | - Experiences of returning to life post-implantation; - Disturbance in relation to body (e.g., sense of physical impairment, scarred body) |
| *Safety & security* | | |
| - Increased personal safety through ability to perceive environmental sounds - Worries and concerns with regards to CI safety | - Fear of losing eyesight; - Having vision important to be safe | - CVI as protection against disease (progression) reinstates sense of safety - receiving information in rehabilitation programs as contribution to sense of safety |
| *Privacy* | | |
| - Gains in privacy through CI; - CI as sign of hearing condition; | x | - Valve sound disclosing private information |
| *Participation* | | |
| - Positive impact of CI on social participation and relationships; - Remaining difficulties caused by situational circumstances, lacking social skills, exclusion from activities due to damage prevention; - CI predominantly perceived relevant for education and employment; - Impact of hearing acculturation on participation; - Experiences of stigmatization and discrimination related to hearing loss or CI | - Invisible condition, overlooked or ignored by others; - Vision essential for social participation | - Social restrictions due to physical symptoms of cardiovascular disease; - Hindering a good social life after implantation due to comorbidities; - CVI relevant for participation in terms of functioning and fulfilling social responsibilities |
| *Justice* | | |
| - Difficulties in accessing healthcare services, e.g., due to long-distance traveling, financial expenses, institutional discrimination | - Cost of glaucoma treatment, e.g., eye drops | - Logistical and financial barriers to accessing healthcare services |
| *Sustainability* | | |
| - Prospect of technological and scientific developments | x | x |
| *Notes.* CI-cochlear implants, GI-glaucoma implants, CVI-cardiovascular implants, n-number of articles, x – no relevant data available | | |

References

Adler, J. M. (2018). Bringing the (disabled) body to personality psychology: A case study of Samantha. *Journal of Personality*, *86*(5), 803–824. https://doi.org/10.1111/jopy.12364

Aloqaili, Y., Arafat, A. S., Almarzoug, A., Alalula, L. S., Hakami, A., Almalki, M., & Alhuwaimel, L. (2019). Knowledge about cochlear implantation: A parental perspective. *Cochlear Implants International*, *20*(2), 74–79. https://doi.org/10.1080/14670100.2018.1548076

Anmyr, L., Olsson, M., Freijd, A., & Larsson, K. (2015). Sense of coherence, social networks, and mental health among children with a cochlear implant. *International Journal of Pediatric Otorhinolaryngology*, *79*(4), 610–615. https://doi.org/10.1016/j.ijporl.2015.02.009

Astin, F., Horrocks, J., McLenachan, J., Blackman, D. J., Stephenson, J., & Closs, S. J. (2017). The impact of transcatheter aortic valve implantation on quality of life: A mixed methods study. *Heart & Lung: The Journal of Critical Care*, *46*(6), 432–438. https://doi.org/10.1016/j.hrtlng.2017.08.005

Athalye, S., Mulla, I., & Archbold, S. (2014). The experiences of adults assessed for cochlear implantation who did not proceed. *Cochlear Implants International*, *15*(6), 301–311. https://doi.org/10.1179/1754762814Y.0000000067

Bat-Chava, Y., & Martin, D. (2002). Sibling relationships for deaf children: The impact of child and family characteristics. *Rehabilitation Psychology*, *47*(1). https://doi.org/10.1037/0090-5550.47.1.73

Baumbusch, J., Lauck, S. B., Achtem, L., O‘Shea, T., Wu, S., & Banner, D. (2018). Understanding experiences of undergoing transcatheter aortic valve implantation: One-year follow-up. *European Journal of Cardiovascular Nursing : Journal of the Working Group on Cardiovascular Nursing of the European Society of Cardiology*, *17*(3), 280–288. https://doi.org/10.1177/1474515117738991

Beattie, R. G., Ritter-Brinton, K., & Snart, F. (2000). A mother and son cochlear implant case study: Making the decision twice. *Advances in Oto-Rhino-Laryngology*, *57*, 141–144. https://doi.org/10.1159/000059222

Berg, S. K., Zwisler, A.‑D., Pedersen, B. D., Haase, K., & Sibilitz, K. L. (2013). Patient experiences of recovery after heart valve replacement: Suffering weakness, struggling to resume normality. *BMC Nursing*, *12*(1), 23. https://doi.org/10.1186/1472-6955-12-23

Bryssinck, L., Vlieger, S. de, François, K., & Bové, T. (2021). Post hoc patient satisfaction with the choice of valve prosthesis for aortic valve replacement: Results of a single-centre survey. *Interactive Cardiovascular and Thoracic Surgery*, *33*(2), 210–217. https://doi.org/10.1093/icvts/ivab069

Chapman, M., & Dammeyer, J. (2017). The Relationship Between Cochlear Implants and Deaf Identity. *American Annals of the Deaf*, *162*(4), 319–332. https://doi.org/10.1353/aad.2017.0030

Chen, S., Karamy, B., Shipp, D., Nedzelski, J., Chen, J., & Lin, V. (2016). Assessment of the psychosocial impacts of cochlear implants on adult recipients and their partners. *Cochlear Implants International*, *17*(2), 90–97. https://doi.org/10.1080/14670100.2015.1102456

Choi, J. E., Hong, S. H., & Moon, I. (2020). Academic Performance, Communication, and Psychosocial Development of Prelingual Deaf Children with Cochlear Implants in Mainstream Schools. *JOURNAL of AUDIOLOGY and OTOLOGY*, *24*(2), 61–70. https://doi.org/10.7874/jao.2019.00346

Cross, V., Shah, P., Glynn, M., & Chidrawar, S. (2009). Regae 5: Can we improve the surgical journey for African-Caribbean patients undergoing glaucoma filtration surgery? Some preliminary findings. *Clinical Ophthalmology*, *3*, 1–12.

Dammeyer, J., Chapman, M., & Marschark, M. (2018). Experience of Hearing Loss, Communication, Social Participation, and Psychological Well-Being Among Adolescents With Cochlear Implants. *American Annals of the Deaf*, *163*(4), 424–439. https://doi.org/10.1353/aad.2018.0027

Dillon, B., & Pryce, H. (2020). What makes someone choose cochlear implantation? An exploration of factors that inform patient decision making. *International Journal of Audiology*, *59*(1), 24–32. https://doi.org/10.1080/14992027.2019.1660917

Dornhoffer, J. (2019). An Otologist’s Experience as a Cochlear Implant PatientThe Power of Neuroplasticity. *JAMA Otolaryngology–Head & Neck Surgery*, *145*(5), 401–402. https://doi.org/10.1001/jamaoto.2019.0054

Finlay, L., & Molano-Fisher, P. (2008). ‘transforming’ self and world: A phenomenological study of a changing lifeworld following a cochlear implant. *Medicine, Health Care, and Philosophy*, *11*(3), 255–267. https://doi.org/10.1007/s11019-007-9116-9

Fitzpatrick, E. M., Jacques, J., & Neuss, D. (2011). Parental perspectives on decision-making and outcomes in pediatric bilateral cochlear implantation. *International Journal of Audiology*, *50*(10), 679–687. https://doi.org/10.3109/14992027.2011.590823

Foo, R. C. M., Lamoureux, E. L., Wong, R. C. K., Ho, S.‑W., Chiang, P. P. C., Rees, G., Aung, T., & Wong, T. T. (2012). Acceptance, Attitudes, and Beliefs of Singaporean Chinese Toward an Ocular Implant for Glaucoma Drug Delivery. *Investigative Ophthalmology & Visual Science*, *53*(13), 8240–8245. https://doi.org/10.1167/iovs.12-10393

Frankel, N. Z. (2014). Surgical aortic valve replacement vs transcatheter aortic valve replacement: A consumer’s perspective regarding data education and transparency of hospitals. *JAMA Internal Medicine*, *174*(4), 495–496. https://doi.org/10.1001/jamainternmed.2013.12829

Goldblat, E., & Most, T. (2018). Cultural Identity of Young Deaf Adults with Cochlear Implants in Comparison to Deaf without Cochlear Implants and Hard-of-Hearing Young Adults. *Journal of Deaf Studies and Deaf Education*, *23*(3), 228–239. https://doi.org/10.1093/deafed/eny007

Hallberg, L. R. M., & Ringdahl, A. (2004). Living with cochlear implants: experiences of 17 adult patients in Sweden. *International Journal of Audiology*, *43*(2), 115–121. https://search.ebscohost.com/login.aspx?direct=true&db=cmedm&AN=15035563&site=ehost-live

Hallberg, L. R. M., Ringdahl, A., Holmes, A., & Carver, C. (2005). Psychological general well-being (quality of life) in patients with cochlear implants: Importance of social environment and age. *International Journal of Audiology*, *44*(12), 706–711. https://doi.org/10.1080/14992020500266852

Hardonk, S., Daniels, S., Desnerck, G., Loots, G., van Hove, G., van Kerschaver, E., Sigurjónsdóttir, H. B., Vanroelen, C., & Louckx, F. (2011). Deaf parents and pediatric cochlear implantation: An exploration of the decision-making process. *American Annals of the Deaf*, *156*(3), 290–304. https://doi.org/10.1353/aad.2011.0027

Hilton, K., Jones, F., Harmon, S., & Cropper, J. (2013). Adolescents’ experiences of receiving and living with sequential cochlear implants: An interpretative phenomenological analysis. *Journal of Deaf Studies and Deaf Education*, *18*(4), 513–531. https://doi.org/10.1093/deafed/ent025

Ibrahim, M. A. (2014). The joy of cochlear implants. *BMJ (Clinical Research Ed.)*, *348*, g2019. https://doi.org/10.1136/bmj.g2019

Incesulu, A., Vural, M., & Erkam, U. (2003). Children with cochlear implants: Parental perspective. *Otology & Neurotology*, *24*(4), 605–611. https://doi.org/10.1097/00129492-200307000-00013

Jeffs, E., Redfern, K., Stanfield, C., Starczewski, H., Stone, S., Twomey, T., & Fortnum, H. (2015). A pilot study to explore the experiences of congenitally or early profoundly deafened candidates who receive cochlear implants as adults. *Cochlear Implants International*, *16*(6), 312–320. https://doi.org/10.1179/1754762815Y.0000000011

Kobosko, J., Jedrzejczak, W. W., Pilka, E., Pankowska, A., & Skarzynski, H. (2015). Satisfaction With Cochlear Implants in Postlingually Deaf Adults and Its Nonaudiological Predictors: Psychological Distress, Coping Strategies, and Self-Esteem. *Ear and Hearing*, *36*(5), 605–618. https://doi.org/10.1097/AUD.0000000000000179

Korteland, N. M., Bras, F. J., van Hout, F. M. A., Kluin, J., Klautz, R. J. M., Bogers, A. J. J. C., & Takkenberg, J. J. M. (2015). Prosthetic aortic valve selection: Current patient experience, preferences and knowledge. *Open Heart*, *2*(1), e000237. https://doi.org/10.1136/openhrt-2015-000237

Kos, M.‑I., Degive, C., Boex, C., & Guyot, J.‑P. (2007). Professional occupation after cochlear implantation. *The Journal of Laryngology and Otology*, *121*(3), 215–218. https://doi.org/10.1017/S0022215106003641

Lauck, S. B., Baumbusch, J., Achtem, L., Forman, J. M., Carroll, S. L., Cheung, A., Ye, J., Wood, D. A., & Webb, J. G. (2016). Factors influencing the decision of older adults to be assessed for transcatheter aortic valve implantation: An exploratory study. *European Journal of Cardiovascular Nursing : Journal of the Working Group on Cardiovascular Nursing of the European Society of Cardiology*, *15*(7), 486–494. https://doi.org/10.1177/1474515115612927

Leigh, I. W., Maxwell-McCaw, D., Bat-Chava, Y., & Christiansen, J. B. (2009). Correlates of psychosocial adjustment in deaf adolescents with and without cochlear implants: A preliminary investigation. *Journal of Deaf Studies and Deaf Education*, *14*(2), 244–259. https://doi.org/10.1093/deafed/enn038

Lytvyn, L., Guyatt, G. H., Manja, V., Siemieniuk, R. A., Zhang, Y., Agoritsas, T., & Vandvik, P. O. (2016). Patient values and preferences on transcatheter or surgical aortic valve replacement therapy for aortic stenosis: A systematic review. *BMJ Open*, *6*(9), e014327. https://doi.org/10.1136/bmjopen-2016-014327

Majorano, M., Maes, M., Morelli, M., Bastianello, T., Guerzoni, L., Murri, A., & Cuda, D. (2018). Socio-emotional adjustment of adolescents with cochlear implants: Loneliness, emotional autonomy, self-concept, and emotional experience at the hospital. *Journal of Child Health Care : For Professionals Working with Children in the Hospital and Community*, *22*(3), 359–370. https://doi.org/10.1177/1367493518757065

Mäki-Torkko, E. M., Vestergren, S., Harder, H., & Lyxell, B. (2015). From isolation and dependence to autonomy - expectations before and experiences after cochlear implantation in adult cochlear implant users and their significant others. *Disability and Rehabilitation*, *37*(6), 541–547. https://doi.org/10.3109/09638288.2014.935490

Mance, J., & Edwards, L. (2012). Deafness-related self-perceptions and psychological well-being in deaf adolescents with cochlear implants. *Cochlear Implants International*, *13*(2), 93–104. https://doi.org/10.1179/1754762811Y.0000000017

Marschark, M., Machmer, E., Spencer, L. J., Borgna, G., Durkin, A., & Convertino, C. (2018). Language and Psychosocial Functioning among Deaf Learners with and without Cochlear Implants. *Journal of Deaf Studies and Deaf Education*, *23*(1), 28–40. https://doi.org/10.1093/deafed/enx035

Marsh, K., Hawken, N., Brookes, E., Kuehn, C., & Liden, B. (2019). Patient-centered benefit-risk analysis of transcatheter aortic valve replacement. *F1000Research*, *8*, 394. https://doi.org/10.12688/f1000research.18796.5

Mauldin, L. (2019). Don’t look at it as a miracle cure: Contested notions of success and failure in family narratives of pediatric cochlear implantation. *Social Science & Medicine (1982)*, *228*(4), 117–125. https://doi.org/10.1016/j.socscimed.2019.03.021

Mehrpoya, A., Jalali, R., Jalali, A., & Namdari, M. (2018). Patient experiences of living with coronary stent. *Journal of Vascular Nursing : Official Publication of the Society for Peripheral Vascular Nursing*, *36*(4), 181–185. https://doi.org/10.1016/j.jvn.2018.07.002

Moog, J. S., Geers, A. E., Gustus, C. H., & Brenner, C. A. (2011). Psychosocial adjustment in adolescents who have used cochlear implants since preschool. *Ear and Hearing*, *32*(1 Suppl), 75S-83S. https://doi.org/10.1097/AUD.0b013e3182014c76

Muigg, F., Weichbold, V. W., Kuehn, H., Seebacher, J., & Galvan, O. (2021). Does Cochlear Implantation Affect Openness-to-Experience in Profound Postlingual Hearing Loss? *The Journal of Deaf Studies and Deaf Education*, *26*(1), 142–146. https://doi.org/10.1093/deafed/enaa031

Newberry, E. (2011). ‘i wish I had known to prepare for that’. Wife, mother, and patient: The impact on family dynamics post-implantation. *Cochlear Implants International*, *12 Suppl 2*, S24-6. https://doi.org/10.1179/146701011X13074645127315

Ng, Z. Y., Lamb, B., Harrigan, S., Archbold, S., Athalye, S., & Allen, S. (2016). Perspectives of adults with cochlear implants on current CI services and daily life. *Cochlear Implants International*, *17 Suppl 1*, 89–93. https://doi.org/10.1080/14670100.2016.1157314

Nijmeijer, H. G., Keijsers, N. M., Huinck, W. J., & Mylanus, E. A. (2021). The effect of cochlear implantation on autonomy, participation and work in postlingually deafened adults: a scoping review. *European Archives of Oto-Rhino-Laryngology : Official Journal of the European Federation of Oto-Rhino-Laryngological Societies (EUFOS) : Affiliated with the German Society for Oto-Rhino-Laryngology - Head and Neck Surgery*, *278*(9), 3135–3154. https://doi.org/10.1007/s00405-020-06490-x

Okubo, S., Takahashi, M., & Kai, I. (2008). How Japanese parents of deaf children arrive at decisions regarding pediatric cochlear implantation surgery: A qualitative study. *Social Science & Medicine*, *66*(12), 2436–2447. https://doi.org/10.1016/j.socscimed.2008.02.013

Olsson, K., Naslund, U., Nilsson, J., & Hornsten, A. (2018). Patients’ experiences of the transcatheter aortic valve implantation trajectory: A grounded theory study. *Nursing Open*, *5*(2), 149–157. https://doi.org/10.1002/nop2.124

Ontario Health (Quality). (2019 Dec). *Minimally Invasive Glaucoma Surgery: A Budget Impact Analysis and Evaluation of Patients’ Experiences, Preferences, and Values* (Ont Health Technol Assess Ser No. 9). https://www.hqontario.ca/Evidence-to-Improve-Care/Health-Technology-Assessment/Journal-Ontario-Health-Technology-Assessment-Series

Ontario Health (Quality). (2020 Nov). *Transcatheter Aortic Valve Implantation in Patients With Severe Aortic Valve Stenosis at Low Surgical Risk: A Health Technology Assessment* (Ont Health Technol Assess Ser No. 14). https://www.hqontario.ca/evidence-to-improve-care/health-technology-assessment/reviewsand-recommendations/transcatheter-aortic-valve-implantation-in-patients-with-severe-aortic-valve-stenosisand-low-surgical-risk

Oterhals, K., Fridlund, B., Nordrehaug, J. E., Haaverstad, R., & Norekvål, T. M. (2013). Adapting to living with a mechanical aortic heart valve: A phenomenographic study. *Journal of Advanced Nursing*, *69*(9), 2088–2098. https://doi.org/10.1111/jan.12076

Preisler, G., Tvingstedt, A.‑L., & Ahlström, M. (2005). Interviews with deaf children about their experiences using cochlear implants. *American Annals of the Deaf*, *150*(3), 260–267. https://doi.org/10.2307/26234729

Punch, R., & Hyde, M. (2011). Social participation of children and adolescents with cochlear implants: A qualitative analysis of parent, teacher, and child interviews. *Journal of Deaf Studies and Deaf Education*, *16*(4), 474–493. https://doi.org/10.1093/deafed/enr001

Rauen, J. A., & Rauen, C. A. (2006). A patient’s bold voice: A journey through cardiac surgery. *AACN Advanced Critical Care*, *17*(2), 133–144.

Sach, T. H., & Whynes, D. K. (2005). Paediatric cochlear implantation: The views of parents. *International Journal of Audiology*, *44*(7). https://doi.org/10.1080/14992020500146500

Sahli, S., & Belgin, E. (2006). Comparison of self-esteem level of adolescents with cochlear implant and normal hearing. *International Journal of Pediatric Otorhinolaryngology*, *70*(9), 1601–1608. https://doi.org/10.1016/j.ijporl.2006.05.003

Schmied Wolfram, Schäfers Hans-Joachim, and Köllner Volker (2015). Lebensqualität oder Lebenserwartung? Kriterien und Informationsquellen für die Entscheidungsfindung bei Patienten im Vorfeld von Aortenklappenoperationen/ Quality of life or life expectancy? Criteria and sources of information in the decision-making of patients undergoing aortic valve surgery. *Z PsychosomMed Psychother*(61), 224–237. https://www.jstor.org/stable/43697847

Singh, U., Kapasi, A., Patel, N., Khandhar, V., & Neupane, A. K. (2019). Expectations and Experience of Children with Unilateral Cochlear Implantation: A Parental Perspective. *Indian Journal of Otolaryngology and Head & Neck Surgery*, *71*(4), 442–448. https://doi.org/10.1007/s12070-019-01611-3

Skaar, E., Ranhoff, A. H., Nordrehaug, J. E., Forman, D. E., & Schaufel, M. A. (2017). Conditions for autonomous choice: A qualitative study of older adults’ experience of decision-making in TAVR. *Journal of Geriatric Cardiology*, *14*(1), 42–48. https://doi.org/10.11909/j.issn.1671-5411.2017.01.007

Spencer, L. J., Tomblin, J. B., & Gantz, B. J. (2012). Growing up with a cochlear implant: Education, vocation, and affiliation. *Journal of Deaf Studies and Deaf Education*, *17*(4), 483–498. https://doi.org/10.1093/deafed/ens024

Steinberg, A., Brainsky, A., Bain, L., Montoya, L., Indenbaum, M., & Potsic, W. (2000). Parental values in the decision about cochlear implantation. *International Journal of Pediatric Otorhinolaryngology*, *55*(2), 99–107. https://doi.org/10.1016/S0165-5876(00)00373-6

Vieira, S. d. S., Dupas, G., & Chiari, B. M. (2018a). Cochlear implant: The family’s perspective. *Cochlear Implants International*, *19*(4), 216–224. https://doi.org/10.1080/14670100.2018.1426406

Vieira, S. d. S., Dupas, G., & Chiari, B. M. (2018b). Effects of cochlear implantation on adulthood. *CoDAS*, *30*(6), e20180001. https://doi.org/10.1590/2317-1782/20182018001

Wald, R. L., & Knutson, J. F. (2000). Deaf cultural identity of adolescents with and without cochlear implants. *The Annals of Otology, Rhinology & Laryngology. Supplement*, *185*, 87–89. https://doi.org/10.1177/0003489400109s1238

Warner-Czyz, A. D., Loy, B., Roland, P. S., & Tobey, E. A. (2013). A comparative study of psychosocial development in children who receive cochlear implants. *Cochlear Implants International*, *14*(5), 266–275. https://doi.org/10.1179/1754762812Y.0000000021

Watson, V., Verschuur, C., & Lathlean, J. (2016). Exploring the experiences of teenagers with cochlear implants. *Cochlear Implants International*, *17*(6), 293–301. https://doi.org/10.1080/14670100.2016.1257472

Wheeler, A., Archbold, S., Gregory, S., & Skipp, A. (2007). Cochlear implants: The young people’s perspective. *Journal of Deaf Studies and Deaf Education*, *12*(3), 303–316. https://doi.org/10.1093/deafed/enm018

Williams, L. (2019). Untreated severe-to-profound hearing loss and the cochlear implant situation: How policy and practice are disabling New Zealand society. *The New Zealand Medical Journal*, *132*(1505), 73–78.
